# Robust-optimal control of rotary inverted pendulum control through fuzzy descriptor-based techniques

**DOI:** 10.1038/s41598-024-56202-2

**Published:** 2024-03-07

**Authors:** Duc-Binh Pham, Quy-Thinh Dao, Ngoc-Tam Bui, Thi-Van-Anh Nguyen

**Affiliations:** 1 Hanoi University of Science and Technology, Hanoi, 11615 Vietnam; 2 Innovative Global Program, Shibaura Institute of Technology, Saitama, 337-8570 Japan

**Keywords:** T–S fuzzy descriptor model, Stability control, Robust-optimal control, Linear matrix inequality, Rotary inverted pendulum, Mechanical engineering, Engineering

## Abstract

Expanding upon the well-established Takagi–Sugeno (T–S) fuzzy model, the T–S fuzzy descriptor model emerges as a robust and flexible framework. This article introduces the development of optimal and robust-optimal controllers grounded in the principles of stability control and fuzzy descriptor systems. By transforming complicated inequalities into linear matrix inequalities (LMI), we establish the essential conditions for controller construction, as delineated in theorems. To substantiate the utility of these controllers, we employ the rotary inverted pendulum as a testbed. Through diverse simulation scenarios, these controllers, rooted in fuzzy descriptor systems, demonstrate their practicality and effectiveness in ensuring the stable control of inverted pendulum systems, even in the presence of uncertainties within the model. This study highlights the adaptability and robustness of fuzzy descriptor-based controllers, paving the way for advanced control strategies in complex and uncertain environments.

## Introduction

The pioneering work of Tomohiro Takagi and Michio Sugeno in 1985 introduced the concept of the T–S fuzzy system^1^, which has since evolved into a highly effective tool for modeling nonlinear systems following extensive research and experimentation^2–4^. Moreover, the T–S fuzzy model has served as the foundation for a plethora of effective control methodologies^5–7^, with the Parallel Distributed Compensator (PDC) method standing out prominently^8–10^. Building upon the robust foundation of the original T–S fuzzy model, Taniguchi^11^ introduced a more versatile modeling approach known as the “fuzzy descriptor system (FDS).” What sets the FDS apart is its flexibility, as it doesn’t necessitate the matrix *E* to be nonsingular, thereby enabling the representation of a broader spectrum of nonlinear systems as fuzzy systems. Importantly, fundamental components such as premise variables and membership functions remain intact within the FDS framework. The primary distinction lies in the computation of fuzzy rules, which varies between the FDS^12,13^, where the number of fuzzy rules may differ on both sides, as opposed to the T–S fuzzy system^1^, where the number of fuzzy rules equations with those on the right side.

In addition to addressing fundamental stability concerns, there exists a realm of more intricate challenges aimed at elevating system quality. Optimal control has emerged as a prevailing approach in the pursuit of heightened system performance. Its notable advantages, such as cost minimization, performance enhancement, and the ability to balance conflicting objectives, contribute to its increasing popularity over non-optimal control methods. The fundamental goal of optimal control is to minimize a predefined cost function, leading to efficient resource allocation and substantial cost savings. In terms of performance, optimal control strategies are meticulously designed to bolster system stability, tracking accuracy, and responsiveness, surpassing the capabilities of non-optimal methodologies. Furthermore, optimal control facilitates a nuanced analysis of trade-offs between competing objectives, offering tailored solutions that align with specific application requirements. Illustrative studies in the realm of optimal control encompass the development of an $H_{\infty }$ nonlinear controller, optimized by solving the Riccati algebraic equation, to regulate oxygen and carbon dioxide levels in blood^14^. Another noteworthy approach involves the fusion of LQR and SMC controllers to create a robust optimal controller for a vehicle suspension system, considering perturbations^15^. Additionally, a novel method integrates neural networks and policy iteration (PI)^16^. This technique employs neural network linear differential inclusion (LDI) to linearize the nonlinear model, while PI finds approximate solutions to the Riccati equation without requiring exact parameter knowledge, presenting promising prospects for addressing intricate nonlinear control challenges. In recent years, optimal control techniques rooted in the Takagi–Sugeno (T–S) fuzzy model have garnered substantial interest. A prevailing approach involves the transformation of a nonlinear dynamic model into a T–S fuzzy counterpart, subsequently employing the Linear Quadratic Regulator (LQR) method to determine the feedback control matrix for each subsystem, as observed in^17^ and^18^. A variation on this theme, as seen in^19^, introduces a Genetic Algorithm (GA) optimization step to fine-tune the weight matrices Q and R. This approach is celebrated for its intuitive simplicity, rendering it highly accessible for theoretical exploration and simulation. Nonetheless, it is vital to acknowledge that optimal solutions, while individually maximizing subsystem performance, may not necessarily culminate in the system’s overall best performance. An alternative avenue for optimal control within T–S fuzzy systems materializes through the fusion of parallel distributed compensation and $H_2$ quadratic finite-horizon integral performance indexing, a strategy known as $H_2$/LMI PDC controllers^20,21^. This technique amalgamates stability-focused Linear Matrix Inequality (LMI) conditions with the hybrid Taguchi genetic algorithm (HTGA) to formulate comprehensive controllers. Its efficacy extends to scenarios involving uncertain models and time-delay systems. Regrettably, when considering T–S descriptor systems, the landscape of optimal control remains relatively unexplored^22^ discusses optimal control in fuzzy descriptor systems with time-varying delay. However, there’s room for further development in applying this algorithm to specific objects. Thus, our investigation into T–S fuzzy descriptor optimal control assumes a pivotal role by comprehensively expounding upon fuzzy controller theory and its practical application to a specific system.

Beyond the pursuit of optimal system performance, there exists a parallel realm of significance - the capacity to adapt and robustly contend with variations in uncertain elements embedded within the model. Notable contributions in this domain, such as^23^ and^24^, have turned their focus toward the enigmatic realm of the uncertain T–S descriptor fuzzy model, devising corresponding control strategies. Concretely, these studies leverage linear matrix inequality (LMI) conditions and harness the potent $H_\infty$ performance metric to illuminate the efficacy of robust fuzzy controllers. A salient observation emerges: while this fusion yields commendable outcomes, it concurrently begets intricacy within the LMIs, subsequently engendering supplementary optimization challenges for the system. Thus, the imperative emerges to scrutinize methods of mitigating and ameliorating the influence of uncertainty, delving into transformative techniques and LMI formulations that circumvent the complexities associated with $H_\infty$-based approaches.

In this paper, we delve into the analysis of stability and the design of fuzzy controllers tailor-made for fuzzy descriptor systems. We initiate this endeavor by presenting the mathematical equation that defines FDS, laying the foundational groundwork for our subsequent investigations. Applying the principles of Lyapunov stability theory, we derive explicit stability conditions for the system. These conditions are then systematically transformed into linear matrix inequalities, rendering them amenable to solution through established mathematical tools, such as Yalmip. From these foundational stability criteria, advanced controllers, including optimal and robust-optimal variants, are introduced to amplify system performance. Our considerations also extend to encompass uncertain components within the fuzzy model, facilitating the formulation of apt control strategies to navigate this inherent uncertainty. To validate the practicality and efficacy of the theories presented, we apply them within the context of a highly nonlinear system, the rotary inverted pendulum. This intricate system, characterized by a drive motor, a rotating arm, and a pendulum bar, presents unique challenges due to the absence of an inherent actuator and its inherent instability, replete with uncertainty. Consequently, the rotary inverted pendulum system serves as an apt testbed for the practical implementation and evaluation of the researched control methodologies. The encouraging simulation results suggest the proposed theory holds immense potential for application beyond the simulated environment. Notably, its ability to combine optimal and robust control within a T–S fuzzy framework makes it highly suitable for real-world objects like two-wheeled balancing mobile robots^25^, Segways^26^, and robot arms^27^, which operate in dynamic and uncertain environments. While T–S control methods have been explored for these domains^25^, there remains significant room for further development and application of the theoretical advancements presented in this article.

The principal achievements of this paper can be summarized as follows:

- Offering stability conditions for the fuzzy descriptor system and consequently formulating controllers utilizing Linear Matrix Inequalities and Parallel Distributed Compensator.
- Enhancing system performance, encompassing both settling time and overall cost, while ensuring stability through the application of an optimal fuzzy controller.
- Leveraging the merits of the optimal controller, devising a robust-optimal controller capable of managing uncertain elements within the model and fortifying the system’s resilience against external disturbances.

## Control design

### Fuzzy descriptor system

Building upon prior research, it has been established that the dynamic equation of a system can be reformulated into a T–S fuzzy system. In order to enhance the system’s descriptive capabilities, the concept of a fuzzy descriptor system is introduced as an extension of the conventional T–S fuzzy system. Consequently, the fuzzy descriptor system can be formally defined as follows:

1 $$\begin{aligned} {\left\{ \begin{array}{ll} \displaystyle \sum _{k=1}^{r_e} v_k(z(t)) E_k {\dot{x}}(t)=\sum _{i=1}^r h_i(z(t))\left( A_i x(t)+B_i u(t)\right) \\ \displaystyle y(t)=\sum _{i=1}^r h_i(z(t)) C_i x(t) \end{array}\right. } \end{aligned}$$

where

- $x(t) \in R^n$, ${y}(t) \in R^q$ and ${u}(t) \in {R}^m$ are the vector of state variables, the output vector, and the input vector, respectively;
- $r_e$ and *r* are the number of fuzzy rules on the left and right sides of the fuzzy descriptor system. The entire system is determined through $r_e*r$ fuzzy rules.
- $z(t) = \{z_1(t), z_2(t), z_3(t),..., z_p(t)\}$ is the vector of known premise variables;
- $h_i(z(t)) \ge 0$ and $v_k(z(t)) \ge 0$ are membership functions of the premise variables that satisfy the convex sum property $\displaystyle \sum _{i=1}^r h_i(z(t))=1$ and $\displaystyle \sum _{k=1}^{r^e} v_k(z(t))=1$.

To facilitate the analysis of stability, Eq. (1) can be reformulated in the form of an augmented system:

2 $$\begin{aligned} {\left\{ \begin{array}{ll} \displaystyle E^*{\dot{x}}^*(t)=\sum _{i=1}^r \sum _{k=1}^{r_e} h_i(z(t))v_k(z(t)) \left( A^*_{ik} x^*(t)+B^*_{i} u(t)\right) \\ \displaystyle y(t)=\sum _{i=1}^r h_i(z(t)) C^*_{i} x^*(t) \end{array}\right. } \end{aligned}$$

where

$$x^*(t) = \begin{bmatrix} x(t) \\ {\dot{x}}(t) \end{bmatrix}$$, $$E^*=\begin{bmatrix} I &{} 0 \\ 0 &{} 0 \\ \end{bmatrix}$$, $$A^*_{ik}=\begin{bmatrix} 0 &{} I \\ A_i &{} -E_k \\ \end{bmatrix}$$, $$B^*_{i}=\begin{bmatrix} 0 \\ B_i \\ \end{bmatrix}$$, $$C^*_{i}=\begin{bmatrix} C_i&0 \end{bmatrix}$$.

### Stability analysis and control design

#### Stability conditions

Ensuring stability is a fundamental prerequisite in controlling any system. Therefore, our initial focus lies in scrutinizing the system’s stability before delving into the intricacies of control design. In line with the approach commonly applied when employing the conventional T–S fuzzy model, we also employ Parallel Distributed Compensation (PDC) as a control strategy for the fuzzy descriptor system. The fuzzy controller of the FDS is derived as:

3 $$\begin{aligned} u=-\sum _{i=1}^r \sum _{k=1}^{r_e} h_i(z(t))v_k(z(t))F_{ik} x(t). \end{aligned}$$

Therefore, this control signal is also written in an augmented form with $$F^*_{ik}=\begin{bmatrix} F_{ik} &{} 0\\ \end{bmatrix}$$:

4 $$\begin{aligned} u=-\sum _{i=1}^r \sum _{k=1}^{r_e} h_i(z(t))v_k(z(t))F^*_{ik} x^*(t). \end{aligned}$$

The closed-loop system is derived from the open-loop system in (2) and the control signal (4):

5 $$\begin{aligned} \begin{aligned} E^*{\dot{x}}^*(t)=\sum _{i=1}^r \sum _{j=1}^r \sum _{k=1}^{r_e} h_i(z(t))h_j(z(t))v_k(z(t))\left( A^*_{ik} -B^*_{i}F^*_{jk}\right) x^*(t). \end{aligned} \end{aligned}$$

##### Theorem 1

If there exist matrices $Z_1$, $Z_3$, $Z_4$ and $M_{ik}$ with appropriate dimension that satisfy

6a $$\begin{aligned}{} & {} {Z}_1^\top ={Z}_1>{0} \end{aligned}$$

6b $$\begin{aligned}{} & {} \begin{bmatrix} -{Z}_3-{Z}_3^\top &{} * \\ {Z}_4^\top +{A}_i {Z}_1-{B}_i {M}_{i k}+{E}_k {Z}_3 &{} -{Z}_4^\top {E}_k^\top -{E}_k {Z}_4 \end{bmatrix}<{0}, \quad h_i \cap v_k \ne \varnothing \text {, } \end{aligned}$$

6c $$\begin{aligned}{} & {} \begin{bmatrix} -2 {Z}_3-2 {Z}_3^\top &{} *\\ 2 {Z}_4^\top +{A}_i {Z}_1 -{B}_i {M}_{j k}+{A}_j {Z}_1 -{B}_j {M}_{i k}+2 {E}_k {Z}_3 &{}\hspace{3mm}-2 {Z}_4^\top {E}_k^\top -2 {E}_k {Z}_4\\ \end{bmatrix} \le 0, \quad i<j \le r \text { s.t. } h_i \cap h_j \cap v_k \ne \varnothing \text {, } \end{aligned}$$

the fuzzy descriptor system in (5) is guaranteed to be stable through the utilization of the fuzzy controller (4). Note that (*) indicates the elements that have been transposed to their symmetric positions $$\begin{bmatrix} x_1 &{} * \\ x_2 &{} x_3 \\ \end{bmatrix} = \begin{bmatrix} x_1 &{} x_2^\top \\ x_2 &{} x_3 \\ \end{bmatrix}$$ and $h_i$, $h_j$, $v_k$ stand for $h_i(z(t))$, $h_j(z(t))$ and $v_k(z(t))$, respectively.

##### Remark 1

This theorem introduces an enhancement to the stability theorem presented in^28^ by introducing the $Z_4$ matrix. In^28^, the matrix $Z_1$ is subjected to numerous constraints, which limits the range of possible solutions for $Z_1$. In contrast, the inclusion of $Z_4$, an arbitrary matrix of appropriate dimension, significantly relaxes the constraints on $Z_1$. This modification makes it easier to find feasible solutions for the LMI conditions.

##### Proof

Consider the non-negative Lyapunov function as:

7 $$\begin{aligned} V(x^*)=x^{*{\top}} E^{*{\top}} X x^*\end{aligned}$$

where $$X = \begin{bmatrix} S_1 &{} 0 \\ S_3 &{} S_4 \\ \end{bmatrix}$$. The Lyapunov function in the above equation resembles the choices made in previous studies on fuzzy descriptor systems^25,29^. Each variation in the selection of the component matrix in *X* results in a distinct solution for LMIs. Careful consideration is required in choosing the form of *X* to strike a balance between solution complexity and the intended level of conservativeness in the LMIs formulation.

Calculating the derivative of $V(x^*)$ yields:

8 $$\begin{aligned} {\dot{V}}(x^*)&=\sum _{i=1}^r \sum _{j=1}^r \sum _{k=1}^{r_e} h_i h_j v_k x^{{*{ \top }}} \left[ \left( A^*_{ik} -B^*_{i}F^*_{jk}\right) ^\top X+X^\top \left( A^*_{ik} -B^*_{i}F^*_{jk}\right) \right] x^*\nonumber \\&=\sum _{i=1}^r \sum _{k=1}^{r_e} h_i^2 v_k x^{{*{ \top }}} \left[ \left( A^*_{ik} -B^*_{i}F^*_{ik}\right) ^\top X+X^\top \left( A^*_{ik} -B^*_{i}F^*_{ik}\right) \right] x^*\nonumber \\&+ 2\sum _{i=1}^r \sum _{i<j} \sum _{k=1}^{r_e} h_i h_j v_k x^{{*{ \top }}} \left[ \left( \frac{A^*_{ik} -B^*_{i}F^*_{jk}+A^*_{jk} -B^*_{j}F^*_{ik}}{2}\right) ^\top X +X^\top \left( \frac{A^*_{ik} -B^*_{i}F^*_{jk}+A^*_{jk} -B^*_{j}F^*_{ik}}{2}\right) \right] x^*\end{aligned}$$

From the above equation, the stability conditions of the fuzzy descriptor system are inferred as follows:

9 $$\begin{aligned}{} & {} E^{{*{ \top }}} X=X^\top E^*\ge 0, \end{aligned}$$

10 $$\begin{aligned}{} & {} G_{iik}^\top X + X^\top G_{iik} < 0, \quad h_i \cap v_k \ne \varnothing \text {, } \end{aligned}$$

11 $$\begin{aligned}{} & {} \left( \frac{G_{ijk}+G_{jik}}{2} \right) ^\top X + X^\top \left( \frac{G_{ijk}+G_{jik}}{2} \right) \le 0, \quad i<j \le r \text { s.t. } h_i \cap h_j \cap v_k \ne \varnothing . \end{aligned}$$

where $$G_{ijk} = A^*_{ik} -B^*_{i}F^*_{jk} = \begin{bmatrix} 0 &{} I \\ A_i-B_iF_{jk} &{} -E_k \\ \end{bmatrix}.$$ Pre and post multiply (9) with $X^{-\top }$ and $X^{-1}$, then:

12 $$\begin{aligned} X^{-\top }E^{{*{ \top }}} = E^*X^{-1} \ge 0. \end{aligned}$$

It means that:

13 $$\begin{aligned} \begin{bmatrix} S_1 &{} 0 \\ S_3 &{} S_4 \\ \end{bmatrix} ^{-\top }\begin{bmatrix} I &{} 0 \\ 0 &{} 0 \\ \end{bmatrix} = \begin{bmatrix} I &{} 0 \\ 0 &{} 0 \\ \end{bmatrix} \begin{bmatrix} S_1 &{} 0 \\ S_3 &{} S_4 \\ \end{bmatrix} ^{-1} \ge 0. \end{aligned}$$

Choose matrix *Z* as:

14 $$\begin{aligned} Z = \begin{bmatrix} Z_1 &{} 0 \\ -Z_3 &{} Z_4 \\ \end{bmatrix} = X^{-1} = \begin{bmatrix} S_1 &{} 0 \\ S_3 &{} S_4 \\ \end{bmatrix} ^{-1} \ge 0 \Rightarrow {\left\{ \begin{array}{ll} Z_1 = S_1^{-1}; \\ Z_3 =S_4^{-1}S_3S_1^{-1}; \\ Z_4 = S_4^{-1}. \end{array}\right. } \end{aligned}$$

From (13) and (14), we have:

15 $$\begin{aligned} \begin{bmatrix} Z_1^\top &{} -Z_3^\top \\ 0 &{} Z_4^\top \\ \end{bmatrix} \begin{bmatrix} I &{} 0 \\ 0 &{} 0 \\ \end{bmatrix} = \begin{bmatrix} I &{} 0 \\ 0 &{} 0 \\ \end{bmatrix} \begin{bmatrix} Z_1 &{} 0 \\ -Z_3 &{} Z_4 \\ \end{bmatrix} \ge 0 \Rightarrow \begin{bmatrix} Z_1^\top &{} 0 \\ 0 &{} 0 \\ \end{bmatrix} = \begin{bmatrix} Z_1 &{} 0 \\ 0 &{} 0 \\ \end{bmatrix}. \end{aligned}$$

Therefore, the condition in (6a) is directly derived from (15). In the same way, consider the condition $G_{iik}^\top X + X^\top G_{iik} < 0$ in (10), we have:

16 $$\begin{aligned} X^{-\top } G_{iik}^\top X X^{-1} + X^{-\top } X^\top G_{iik}X^{-1} < 0 \end{aligned}$$

17 $$\begin{aligned} \begin{aligned} \Leftrightarrow&\begin{bmatrix} Z_1^\top &{} -Z_3^\top \\ 0 &{} Z_4^\top \\ \end{bmatrix} \begin{bmatrix} 0 &{} A_i^\top -F_{ik}^\top B_i^\top \\ I &{} -E_k^\top \\ \end{bmatrix} + \begin{bmatrix} 0 &{} I \\ A_i-B_iF_{ik} &{} -E_k \\ \end{bmatrix} \begin{bmatrix} Z_1 &{} 0 \\ -Z_3 &{} Z_4 \\ \end{bmatrix} < 0 \end{aligned} \end{aligned}$$

Then

18 $$\begin{aligned} \begin{bmatrix} -Z_3-Z_3^\top &{} *\\ Z_4^\top + A_iZ_1 - B_iF_{ik}Z_1 + E_kZ_3 &{} -Z_4^\top E_k^\top - E_kZ_4 \\ \end{bmatrix} < 0 \end{aligned}$$

Let $M_{ik} = F_{ik}Z_1$, we can eliminate the BMI term in (18):

19 $$\begin{aligned} \begin{bmatrix} -Z_3-Z_3^\top &{} *\\ Z_4^\top + A_iZ_1 - B_iM_{ik} + E_kZ_3 &{} -Z_4^\top E_k^\top - E_kZ_4 \\ \end{bmatrix} < 0 \end{aligned}$$

Condition (6c) can also be inferred from (11):

20 $$\begin{aligned} \left( \frac{G_{ijk}+G_{jik}}{2} \right) ^\top X + X^\top \left( \frac{G_{ijk}+G_{jik}}{2} \right) \le 0 \end{aligned}$$

$$\begin{aligned} \begin{aligned}{}&\Rightarrow \begin{bmatrix} Z_1^\top &{} -Z_3^\top \\ 0 &{} Z_4^\top \\ \end{bmatrix} \begin{bmatrix}0 &{} A_i^\top + A_j^\top -F_{jk}^\top B_i^\top -F_{ik}^\top B_j^\top \\ 2I &{} -2E_k^\top \\ \end{bmatrix} + \begin{bmatrix} 0 &{} 2I \\ A_i+A_j-B_iF_{jk}-B_jF_{ik} &{} -2E_k \\ \end{bmatrix} \begin{bmatrix} Z_1 &{} 0 \\ -Z_3 &{} Z_4 \\ \end{bmatrix} \le 0 \end{aligned} \end{aligned}$$

With $M_{ik} = F_{ik}Z_1$ and $M_{jk} = F_{jk}Z_1$, we obtain condition (6c):

$$\begin{aligned} \begin{aligned} \begin{bmatrix} -2 {Z}_3-2 {Z}_3^\top &{} *\\ 2 {Z}_4^\top +{A}_i {Z}_1 -{B}_i {M}_{j k} +{A}_j {Z}_1 -{B}_j {M}_{i k}+2 {E}_k {Z}_3 \hspace{5mm}&{} -2 {Z}_4^\top {E}_k^\top -2 {E}_k {Z}_4 \end{bmatrix} \le {0} \end{aligned} \end{aligned}$$

$$\square$$

#### Optimal fuzzy control

To expedite the response time of state variables and optimize the system’s performance, we can utilize optimal fuzzy control. Consider the quadratic cost function with $W\ge 0$ and $R>0$:

21 $$\begin{aligned} J=\int _{0}^{\infty }\left( {y}^\top (t)Wy(t)+u^\top (t)Ru(t) \right) dt. \end{aligned}$$

where

$$\begin{aligned} y(t) = \sum _{i=1}^r h_i(z(t)) C_i^*x^*(t) \end{aligned}$$

The presented quadratic cost function bears a resemblance to the cost function utilized in the LQR (Linear Quadratic Regulator) method, particularly when the output *y*(*t*) encompasses the entire state. Nevertheless, the path to resolving the optimization problem for these two methods diverges significantly: LQR necessitates the solution of the Riccati equation to determine the optimal solution, whereas the T–S descriptor fuzzy optimal method endeavors to minimize costs by constructing an upper bound on the cost function. By formulating the relationship between the cost function and its upper bound using the LMI condition, the minimization of the upper bound indirectly leads to the minimization of the cost function value. Subsequently, the upcoming theorem will shed light on the conditions in optimal fuzzy control.

##### Theorem 2

If there exist matrices $Z_1$, $Z_3$, $Z_4$ and $M_{ik} = F_{ik}Z_1$ that satisfy the following conditions:

22a $$\begin{aligned}{} & {} {Z}_1^\top ={Z}_1>{0}, \end{aligned}$$

22b $$\begin{aligned}{} & {} \begin{bmatrix} \lambda &{} {x}^\top (0) \\ {x} (0) &{} Z_1 \\ \end{bmatrix} > 0, \end{aligned}$$

22c $$\begin{aligned}{} & {} {\begin{bmatrix} -{Z}_3-{Z}_3^\top &{} * &{} * &{} *\\ {Z}_4^\top +{A}_i {Z}_1-{B}_i {M}_{i k}+{E}_k {Z}_3 &{} -{Z}_4^\top {E}_k^\top -{E}_k {Z}_4 &{} * &{} *\\ C_iZ_1 &{} 0 &{} -W^{-1} &{} * \\ -M_{ik} &{} 0 &{} 0 &{} -R^{-1} \end{bmatrix}<{0},}\quad h_i \cap v_k \ne \varnothing \text {, } \end{aligned}$$

22d $$\begin{aligned}{} & {} \begin{aligned} \begin{bmatrix} -2 {Z}_3-2 {Z}_3^\top &{} * &{} * &{} * &{} * &{} *\\ 2 {Z}_4^\top +{A}_i {Z}_1 -{B}_i {M}_{j k}+{A}_j {Z}_1 -{B}_j {M}_{i k}+2 {E}_k {Z}_3 &{} -2 {Z}_4^\top {E}_k^\top -2 {E}_k {Z}_4 &{} * &{} * &{} * &{} *\\ C_iZ_1 &{} 0 &{} -W^{-1} &{} * &{} * &{} *\\ -M_{jk} &{} 0 &{} 0 &{} -R^{-1} &{} * &{} *\\ C_jZ_1 &{} 0 &{} 0 &{} 0 &{} -W^{-1} &{} *\\ -M_{ik} &{} 0 &{} 0 &{} 0 &{} 0 &{} -R^{-1} \end{bmatrix}< {0}, \\ i<j \le r \text { s.t. } h_i \cap h_j \cap v_k \ne \varnothing \text {, } \end{aligned} \end{aligned}$$

the cost function (21) will be optimized through minimizing the upper-bound $\lambda$, and the closed-loop descriptor system (5) can be stabilized via PDC controller.

Adjusting parameters such as $\lambda$ or the coefficients of matrices *W* and *R* has a direct influence on the control signal. Improperly selected coefficients can lead to a substantial control signal magnitude or, more critically, impede the determination of $F_{ik}$ by preventing the discovery of a solution that complies with the LMIs conditions.

##### Proof

Define new variable ${\hat{y}}(t)$:

23 $$\begin{aligned} {\hat{y}}(t) = \begin{bmatrix} y(t) \\ u(t) \\ \end{bmatrix} = \sum _{i=1}^r \sum _{k=1}^{r_e} h_i(z(t))v_k(z(t)) \begin{bmatrix} C_i^*\\ F^*_{ik} \end{bmatrix} x^*(t) \end{aligned}$$

With the above ${\hat{y}}(t)$, the cost function from (21) can be rewritten as:

24 $$\begin{aligned} J=\int _{0}^{\infty }{\hat{y}}^\top (t) \begin{bmatrix} W &{} 0 \\ 0 &{} R \\ \end{bmatrix} {\hat{y}}(t)dt \end{aligned}$$

where $W\ge 0$ and $R>0$. Assume that there exists a matrix *X* satisfying

25 $$\begin{aligned} G_{iik}^\top X + X^\top G_{iik} + \begin{bmatrix} {C_i}^{*{\top}} &{} {-F_{ik}}^{*{\top}} \\ \end{bmatrix} \begin{bmatrix} W &{} 0 \\ 0 &{} R \\ \end{bmatrix} \begin{bmatrix} C^*_i \\ -F_{ik}^*\end{bmatrix} < 0, \end{aligned}$$

26 $$\begin{aligned} (G_{ijk}+G_{jik})^\top X + X^\top (G_{ijk}+G_{jik}) + \begin{bmatrix} {C^*_i}^\top&{-F_{jk}}^{*{\top}} \end{bmatrix} \begin{bmatrix} W &{} 0 \\ 0 &{} R \\ \end{bmatrix} \begin{bmatrix} C^*_i \\ -F_{jk}^*\end{bmatrix} + \begin{bmatrix} {C^*_j}^\top &{} {-F_{ik}}^{*{\top}} \\ \end{bmatrix} \begin{bmatrix} W &{} 0 \\ 0 &{} R \\ \end{bmatrix} \begin{bmatrix} C^*_j \\ -F_{ik}^*\end{bmatrix} < 0, \end{aligned}$$

then we have:

27 $$\begin{aligned}{} & {} G_{iik}^\top X + X^\top G_{iik} < 0, \quad h_i \cap v_k \ne \varnothing \text {, } \end{aligned}$$

28 $$\begin{aligned}{} & {} ( G_{ijk}+G_{jik})^\top X + X^\top (G_{ijk}+G_{jik})< 0, \quad i<j \le r \text { s.t. } h_i \cap h_j \cap v_k \ne \varnothing . \end{aligned}$$

It’s evident that the conditions stated above coincide with the conditions outlined in Theorem 1. Consequently, if we are able to identify a matrix *X* that satisfies both (25) and (26), it guarantees the global asymptotic stability of the system. In the realm of optimal control problems, the primary objective is to minimize the cost function *J* achieving the smallest possible value. To accomplish this, rather than pursuing the optimal solution through the Riccati algebraic equation, an additional upper bound is introduced to constrain the value of *J*. Consider the Lyapunov function as in (7), we have the following derivative of *V*:

29 $$\begin{aligned} {\dot{V}}(x^*)= & {} \sum _{i=1}^r \sum _{k=1}^{r_e} h_i^2 v_kx^{{*{ \top }}} \left[ G_{iik}^\top X+X^\top G_{iik}\right] x^*\nonumber \\{} & {} + \sum _{i=1}^r \sum _{i<j} \sum _{k=1}^{r_e} h_i h_j v_k x^{{*{ \top }}} \left[ (G_{ijk}+G_{jik})^\top X + X^\top (G_{ijk}+G_{jik})\right] x^*. \end{aligned}$$

Utilizing conditions in (25) and (26) yields:

30 $$\begin{aligned}{}&{\dot{V}}(x^*)<-\sum _{i=1}^r \sum _{k=1}^{r_e} h_i^2 v_kx^{{*{ \top }}} \begin{bmatrix} {C^*_i}^\top {-F_{ik}}^{*{\top}} \end{bmatrix} \begin{bmatrix} W &{} 0 \\ 0 &{} R \\ \end{bmatrix} \begin{bmatrix} C^*_i \\ -F_{ik}^*\end{bmatrix} x^*-\sum _{i=1}^r \sum _{i<j} \sum _{k=1}^{r_e} h_ih_j v_kx^{{*{ \top }}} \begin{bmatrix} {C^*_i}^\top&{-F_{jk}}^{*{\top}} \end{bmatrix} \begin{bmatrix} W &{} 0 \\ 0 &{} R \\ \end{bmatrix} \begin{bmatrix} C^*_i \\ -F_{jk}^*\end{bmatrix} x^*\nonumber \\&-\sum _{i=1}^r \sum _{i<j} \sum _{k=1}^{r_e} h_ih_j v_kx^{{*{ \top }}} \begin{bmatrix} {C^*_j}^\top&{-F_{ik}}^{*{\top}} \end{bmatrix} \begin{bmatrix} W &{} 0 \\ 0 &{} R \\ \end{bmatrix} \begin{bmatrix} C^*_j \\ -F_{ik}^*\end{bmatrix} x^*\end{aligned}$$

$$\begin{aligned}{}&\le -x^{{*{ \top }}} \left\{ \sum _{i=1}^r \sum _{k=1}^{r_e}h_i^2 v_k \begin{bmatrix} {C^*_i}^\top&{-F_{ik}}^{*{\top}} \end{bmatrix} \begin{bmatrix} W &{} 0 \\ 0 &{} R \\ \end{bmatrix} \begin{bmatrix} C^*_i \\ -F_{ik}^*\end{bmatrix} \right\} x^*-x^{{*{ \top }}} \left\{ \sum _{i=1}^r \sum _{i<j} \sum _{k=1}^{r_e} h_ih_j v_k \begin{bmatrix} {C^*_i}^\top&{-F_{jk}}^{*{\top}} \end{bmatrix} \begin{bmatrix} W &{} 0 \\ 0 &{} R \\ \end{bmatrix} \begin{bmatrix} C^*_j \\ -F_{ik}^*\end{bmatrix} \right\} x^*\\&-x^{{*{ \top }}} \left\{ \sum _{i=1}^r \sum _{i<j} \sum _{k=1}^{r_e} h_ih_j v_k \begin{bmatrix} {C^*_j}^\top&{-F_{ik}}^{*{\top}} \end{bmatrix} \begin{bmatrix} W &{} 0 \\ 0 &{} R \\ \end{bmatrix} \begin{bmatrix} C^*_i \\ -F_{jk}^*\end{bmatrix} \right\} x^*\end{aligned}$$

$$\begin{aligned} \begin{aligned} \qquad&=-x^{{*{ \top }}} \left\{ \sum _{i=1}^r \sum _{j=1}^r \sum _{k=1}^{r_e} h_ih_j v_k \begin{bmatrix} {C^*_i}^\top&{-F_{ik}}^{*{\top}} \end{bmatrix} \begin{bmatrix} W &{} 0 \\ 0 &{} R \\ \end{bmatrix} \begin{bmatrix} C^*_j \\ -F_{jk}^*\end{bmatrix} \right\} x^*\\ {}&=-x^{{*{ \top }}} \left\{ \left( \sum _{i=1}^r \sum _{k=1}^{r_e} h_i v_k \begin{bmatrix} {C^*_i}^\top&{-F_{ik}}^{*{\top}} \end{bmatrix}\right) \begin{bmatrix} W &{} 0 \\ 0 &{} R \\ \end{bmatrix} \left( \sum _{i=1}^r \sum _{k=1}^{r_e} h_i v_k \begin{bmatrix} C^*_i &{} -F_{ik}^*\\ \end{bmatrix} \right) \right\} x^*\\ {}&=-{\hat{y}}^\top \begin{bmatrix} W &{} 0 \\ 0 &{} R \\ \end{bmatrix} {\hat{y}} \end{aligned} \end{aligned}$$

Therefore we have:

31 $$\begin{aligned} \frac{d}{dt}\left( x^{{*{ \top }}} E^{{*{ \top }}} X x^*\right) < -{\hat{y}}^\top \begin{bmatrix} W &{} 0 \\ 0 &{} R \\ \end{bmatrix} {\hat{y}}. \end{aligned}$$

Integrating both side from 0 to $\infty$, we get:

32 $$\begin{aligned} J=\int _{0}^{\infty }{\hat{y}}^\top (t) \begin{bmatrix} W &{} 0 \\ 0 &{} R \\ \end{bmatrix} {\hat{y}}(t)dt< -\left. x^{{*{ \top }}} (t) E^{{*{ \top }}} X x^*(t)\right| ^\infty _0 \Rightarrow J=\int _{0}^{\infty }{\hat{y}}^\top (t) \begin{bmatrix} W &{} 0 \\ 0 &{} R \\ \end{bmatrix} {\hat{y}}(t)dt < x^{{*{ \top }}} (0) E^{{*{ \top }}} X x^*(0). \end{aligned}$$

Examining Eq. (32), it becomes evident that the cost function *J* possesses an upper bound. Nonetheless, the matrix *X* that meets the stipulated conditions represents just one feasible solution, not necessarily the optimal one. To clearly define this upper limit, we introduce an additional variable, denoted as $\lambda$. Through the incorporation of $\lambda$, it is capable of transforming this ambiguous condition into a well-defined LMI condition.

33 $$\begin{aligned} J< x^{{*{ \top }}} (0) E^{{*{ \top }}} X x^*(0) < \lambda . \end{aligned}$$

With the definition of ${E^*}$ and (14), we have:

34 $$\begin{aligned} \begin{aligned}{}&x^{{*{ \top }}} (0) \begin{bmatrix} I &{} 0 \\ 0 &{} 0 \\ \end{bmatrix} \begin{bmatrix} Z_1^{-1} &{} 0 \\ Z_4^{-1}Z_3Z_1^{-1} &{} Z_4^{-1} \\ \end{bmatrix} x^*(0)< \lambda \\&\Leftrightarrow \begin{bmatrix} x^\top (0) &{} {\dot{x}}^\top (0) \\ \end{bmatrix} \begin{bmatrix} Z_1^{-1} &{} 0 \\ 0 &{} 0 \\ \end{bmatrix} \begin{bmatrix} x(0) \\ {\dot{x}}(0)\\ \end{bmatrix}< \lambda \\&\Leftrightarrow x^\top (0)Z_1^{-1}x(0) < \lambda . \end{aligned} \end{aligned}$$

From Schur complement, the above condition can be transformed into LMI condition:

35 $$\begin{aligned} \begin{bmatrix} \lambda &{} x^\top (0) \\ x(0) &{} Z_1 \\ \end{bmatrix} >0. \end{aligned}$$

Recall the condition in (25) and apply the Schur complement. We have the following LMI:

36 $$\begin{aligned} \begin{bmatrix} \left( A^*_{ik} -B^*_{i}F^*_{ik}\right) ^\top X +X^\top \left( A^*_{ik} -B^*_{i}F^*_{ik}\right) &{} {C^*_i}^\top &{} {-F^*_{ik}}^\top \\ {C^*_i} &{} -W^{-1} &{} 0\\ -F^*_{ik} &{} 0 &{} -R^{-1}\\ \end{bmatrix} < 0. \end{aligned}$$

Multiplying diag$(X^{-\top },I,I)$ to the left and diag$(X^{-1},I,I)$ to the right of the above inequality yields:

$$\begin{aligned} \begin{bmatrix} X^{-\top } &{} 0 &{} 0 \\ 0 &{} I &{} 0 \\ 0 &{} 0 &{} I \\ \end{bmatrix} \begin{bmatrix} \left( A^*_{ik} -B^*_{i}F^*_{ik}\right) ^\top X +X^\top \left( A^*_{ik} -B^*_{i}F^*_{ik}\right) &{} {C^*_i}^\top &{} {-F^*_{ik}}^\top \\ {C^*_i} &{} -W^{-1} &{} 0 \\ -F^*_{ik} &{} 0 &{} -R^{-1} \\ \end{bmatrix} \begin{bmatrix} X^{-1} &{} 0 &{} 0 \\ 0 &{} I &{} 0 \\ 0 &{} 0 &{} I \\ \end{bmatrix} < 0. \end{aligned}$$

Based on Theorem 1, the following LMI condition is directly inferred as:

37 $$\begin{aligned} \begin{bmatrix} -{Z}_3-{Z}_3^\top &{} * &{} * &{} *\\ {Z}_4^\top +{A}_i {Z}_1 -{B}_i {M}_{i k}+{E}_k {Z}_3 &{} -{Z}_4^\top {E}_k^\top -{E}_k {Z}_4 &{} * &{} *\\ C_iZ_1 &{} 0 &{} -W^{-1} &{} * \\ -M_{ik} &{} 0 &{} 0 &{} -R^{-1} \end{bmatrix}<{0}. \end{aligned}$$

Similarly, the last condition (22d) is inferred from inequality (26):

38 $$\begin{aligned} \begin{bmatrix} \left( A^*_{ik} -B^*_{i}F^*_{jk}+A^*_{jk} -B^*_{j}F^*_{ik}\right) ^\top X +X^\top \left( A^*_{ik} -B^*_{i}F^*_{jk}+A^*_{jk} -B^*_{j}F^*_{ik}\right) &{} {C^*_i}^\top &{} {F^*_{jk}}^\top &{} {C^*_j}^\top &{} {F^*_{ik}}^\top \\ {C^*_i} &{} -W^{-1} &{} 0 &{} 0 &{} 0\\ -F^*_{jk} &{} 0 &{} -R^{-1} &{} 0 &{} 0\\ {C^*_j} &{} 0 &{} 0 &{} -W^{-1} &{} 0\\ -F^*_{ik} &{} 0 &{} 0 &{} 0 &{} -R^{-1}\\ \end{bmatrix} < 0. \end{aligned}$$

We have:

39 $$\begin{aligned} \begin{bmatrix} -2 {Z}_3-2 {Z}_3^\top &{} * &{} * &{} * &{} * &{} *\\ 2 {Z}_4^\top +{A}_i {Z}_1 -{B}_i {M}_{j k}+{A}_j {Z}_1 -{B}_j {M}_{i k}+2 {E}_k {Z}_3 &{} -2 {Z}_4^\top {E}_k^\top -2 {E}_k {Z}_4 &{} * &{} * &{} * &{} *\\ C_iZ_1 &{} 0 &{} -W^{-1} &{} * &{} * &{} *\\ -M_{jk} &{} 0 &{} 0 &{} -R^{-1} &{} * &{} *\\ C_jZ_1 &{} 0 &{} 0 &{} 0 &{} -W^{-1} &{} *\\ -M_{ik} &{} 0 &{} 0 &{} 0 &{} 0 &{} -R^{-1}\\ \end{bmatrix} < {0}. \end{aligned}$$

$$\square$$

#### Robust-optimal fuzzy control

The preceding section delved into the domain of optimal fuzzy control, primarily concerned with formulating control strategies to optimize system performance. This approach inherently relies on a precisely defined model of the system. However, practical systems frequently exhibit uncertainties and variations that challenge this idealized representation. In this section, the system will be represented using the uncertain T–S fuzzy model, taking into account uncertain components. From this fuzzy model, a robust fuzzy control strategy is then developed to ensure both system stability and resilience in the presence of model uncertainties and external disturbances. Robust fuzzy control serves as a safeguard, enabling controllers to adeptly manage deviations from the ideal model. Incorporating both optimal fuzzy control and robust fuzzy control techniques, the synthesis yields robust-optimal fuzzy control, harnessing the merits of both methods to create a unified controller of exceptional capability.

Consider the uncertain T–S descriptor model:

40 $$\begin{aligned} \begin{aligned} \sum _{k=1}^{r_e} v_k(z(t)) \left( E_k+\Delta E_k \right) {\dot{x}}(t)=\sum _{i=1}^r h_i(z(t))\left\{ (A_i+\Delta A_i) x(t)+(B_i+\Delta B_i) u(t) \right\} \end{aligned} \end{aligned}$$

where uncertain matrices are defined as:

$$\begin{aligned} \begin{aligned} \Delta A_i&= H_{ai}\Delta a_i (t) W_{ai}, \quad \Delta a_i^\top (t) \Delta a_i (t) \le I\\ \Delta B_i&= H_{bi}\Delta b_i (t) W_{bi}, \quad \Delta b_i^\top (t) \Delta b_i (t) \le I \\ \Delta E_k&= H_{ek}\Delta e_k (t) W_{ek}, \quad \Delta e_k^\top (t) \Delta e_k (t) \le I \end{aligned} \end{aligned}$$

The system can also be reformulated into an augmented form:

41 $$\begin{aligned} E^*{\dot{x}}^*(t)=\sum _{i=1}^r \sum _{k=1}^{r_e} h_i(z(t))v_k(z(t))\left( A^*_{ik} x^*(t)+B^*_{i} u(t)\right) \end{aligned}$$

42 $$\begin{aligned} u(t)=-\sum _{i=1}^r \sum _{k=1}^{r_e} h_i(z(t))v_k(z(t))F^*_{ik} x^*(t) \end{aligned}$$

where $$x^*(t) = \begin{bmatrix} x(t) \\ {\dot{x}}(t) \\ \end{bmatrix}$$, $$E^*=\begin{bmatrix} I &{} 0 \\ 0 &{} 0 \\ \end{bmatrix}, B^*_{i}= \begin{bmatrix} 0 \\ B_i+ \Delta B_i \\ \end{bmatrix}$$, $$A^*_{ik}=\begin{bmatrix} 0 &{} I \\ A_i+\Delta A_i &{} -E_k-\Delta E_k \\ \end{bmatrix}, F^*_{ik}= \begin{bmatrix} F_{ik} &{} 0 \\ \end{bmatrix}$$

##### Theorem 3

The uncertain T–S fuzzy descriptor system (41) can be stabilized and mitigate the impact of uncertain components through the fuzzy controller (42) if there exist matrices $Z_1$, $Z_3$, $Z_4$, $M_{ik} = F_{ik}Z_1$ and diagonal positive definite matrices $\tau _a$, $\tau _b$ and $\tau _e$ such that

43a $$\begin{aligned}{} & {} {Z}_1^\top ={Z}_1>{0}, \end{aligned}$$

43b $$\begin{aligned}{} & {} \begin{bmatrix} -{Z}_3-{Z}_3^\top &{} * &{} * &{} * &{} *\\ {Z}_4^\top +{A}_i {Z}_1 -{B}_i {M}_{j k}+{E}_k {Z}_3 &{} -{Z}_4^\top {E}_k^\top -{E}_k {Z}_4 +\tau _aH_{ai}H_{ai}^\top +\tau _bH_{bi}H_{bi}^\top +\tau _eH_{ek}H_{ek}^\top &{} * &{} * &{} *\\ W_{ai}Z_1 &{} 0 &{} -\tau _a &{} * &{} *\\ -W_{bi}M_{jk} &{} 0 &{} 0 &{} -\tau _b &{} *\\ W_{ek}Z_3 &{} -W_{ek}Z_4 &{} 0 &{} 0 &{} -\tau _e \end{bmatrix} <{0} \end{aligned}$$

##### Proof

According to the Theorem 1, we can have sufficient stability conditions for uncertain fuzzy system:

44 $$\begin{aligned}{} & {} {Z}_1^\top ={Z}_1>{0}, \end{aligned}$$

45 $$\begin{aligned}{} & {} \begin{bmatrix} -{Z}_3-{Z}_3^\top &{} * \\ {Z}_4^\top +{A}_i {Z}_1-{B}_i {M}_{j k} +{E}_k {Z}_3+\Delta {A}_i {Z}_1 -\Delta {B}_i {M}_{j k}+\Delta {E}_k {Z}_3 &{} -{Z}_4^\top {E}_k^\top -{E}_k {Z}_4 -{Z}_4^\top \Delta {E}_k^\top -\Delta {E}_k {Z}_4 \end{bmatrix}<{0}. \end{aligned}$$

In the given condition, it becomes apparent that the uncertain terms $\Delta A_i$, $\Delta B_i$, and $\Delta E_k$ remain unspecified. Consequently, there arises a necessity to reformulate this condition to eliminate the influence of these uncertain elements. The indeterminate terms can be isolated as follows:

46 $$\begin{aligned} \begin{bmatrix} -{Z}_3-{Z}_3^\top &{} * \\ {Z}_4^\top +{A}_i {Z}_1-{B}_i {M}_{j k}+{E}_k {Z}_3 &{} -{Z}_4^\top {E}_k^\top -{E}_k {Z}_4 \end{bmatrix} +\begin{bmatrix} 0 &{} * \\ \Delta {A}_i {Z}_1-\Delta {B}_i {M}_{j k}+\Delta {E}_k {Z}_3 &{} -Z_4^\top \Delta {E}_k^\top -\Delta {E}_k {Z}_4 \end{bmatrix}<{0}. \end{aligned}$$

Drawing upon the definitions of $\Delta A_i$, $\Delta B_i$, and $\Delta E_k$, the second matrix in the preceding inequality can be represented as:

47 $$\begin{aligned} \begin{aligned} \begin{bmatrix} 0 &{} * \\ \Delta {A}_i {Z}_1-\Delta {B}_i {M}_{j k}+\Delta {E}_k {Z}_3 &{} -Z_4^\top \Delta {E}_k^\top -\Delta {E}_k {Z}_4\\ \end{bmatrix}&= \begin{bmatrix} 0 &{} 0 &{} 0 \\ H_{ai} &{} H_{bi} &{} H_{ek} \\ \end{bmatrix} \begin{bmatrix} \Delta a_i &{} 0 &{} 0 \\ 0 &{} \Delta b_i &{} 0 \\ 0 &{} 0 &{} \Delta e_k \\ \end{bmatrix} \begin{bmatrix} W_{ai}Z_1 &{} 0\\ -W_{bi}M_{jk} &{} 0 \\ W_{ek}Z_3 &{} -W_{ek}Z_4 \\ \end{bmatrix}\\ {}&+ \begin{bmatrix} Z_1^\top W_{ai}^\top &{} -M_{jk}^\top W_{bi}^\top &{} Z_3^\top W_{ek}^\top \\ 0 &{} 0 &{} -Z_4^\top W_{ek}^\top \\ \end{bmatrix} \begin{bmatrix} \Delta a_i^\top &{} 0 &{} 0 \\ 0 &{} \Delta b_i^\top &{} 0 \\ 0 &{} 0 &{} \Delta e_k^\top \\ \end{bmatrix} \begin{bmatrix} 0 &{} H_{ai}^\top \\ 0 &{} H_{bi}^\top \\ 0 &{} H_{ek}^\top \\ \end{bmatrix} \end{aligned} \end{aligned}$$

Utilize the property in^30^:

48 $$\begin{aligned} {\left\{ \begin{array}{ll} T = T^\top > 0, \\ X^\top Y + Y^\top X \le X^\top T X + Y^\top T^{-1} Y. \end{array}\right. } \end{aligned}$$

and choose $$T = \begin{bmatrix} \tau _a &{} 0 &{} 0 \\ 0 &{} \tau _b &{} 0 \\ 0 &{} 0 &{} \tau _e \\ \end{bmatrix}$$ where $\tau _a, \tau _b, \tau _e$ are diagonal positive definite matrices, we have:

49 $$\begin{aligned} \begin{aligned}{}&\begin{bmatrix} 0 &{} 0 &{} 0 \\ H_{ai} &{} H_{bi} &{} H_{ek} \end{bmatrix} \begin{bmatrix} \Delta a_i &{} 0 &{} 0 \\ 0 &{} \Delta b_i &{} 0 \\ 0 &{} 0 &{} \Delta e_k \\ \end{bmatrix} \begin{bmatrix} W_{ai}Z_1 &{} 0\\ -W_{bi}M_{jk} &{} 0 \\ W_{ek}Z_3 &{} -W_{ek}Z_4 \\ \end{bmatrix} +\\&\begin{bmatrix} Z_1^\top W_{ai}^\top &{} -M_{jk}^\top W_{bi}^\top &{} Z_3^\top W_{ek}^\top \\ 0 &{} 0 &{} -Z_4^\top W_{ek}^\top \\ \end{bmatrix} \begin{bmatrix} \Delta a_i^\top &{} 0 &{} 0 \\ 0 &{} \Delta b_i^\top &{} 0 \\ 0 &{} 0 &{} \Delta e_k^\top \\ \end{bmatrix} \begin{bmatrix} 0 &{} H_{ai}^\top \\ 0 &{} H_{bi}^\top \\ 0 &{} H_{ek}^\top \\ \end{bmatrix} \\&\le \begin{bmatrix} 0 &{} 0 &{} 0 \\ H_{ai} &{} H_{bi} &{} H_{ek} \\ \end{bmatrix} \begin{bmatrix} \Delta a_i &{} 0 &{} 0 \\ 0 &{} \Delta b_i &{} 0 \\ 0 &{} 0 &{} \Delta e_k \\ \end{bmatrix} \begin{bmatrix} \tau _a &{} 0 &{} 0 \\ 0 &{} \tau _b &{} 0 \\ 0 &{} 0 &{} \tau _e \\ \end{bmatrix} \begin{bmatrix} \Delta a_i^\top &{} 0 &{} 0 \\ 0 &{} \Delta b_i^\top &{} 0 \\ 0 &{} 0 &{} \Delta e_k^\top \\ \end{bmatrix} \begin{bmatrix} 0 &{} H_{ai}^\top \\ 0 &{} H_{bi}^\top \\ 0 &{} H_{ek}^\top \\ \end{bmatrix} \\&+ \begin{bmatrix} Z_1^\top W_{ai}^\top &{} -M_{jk}^\top W_{bi}^\top &{} Z_3^\top W_{ek}^\top \\ 0 &{} 0 &{} -Z_4^\top W_{ek}^\top \\ \end{bmatrix} \begin{bmatrix} \tau _a &{} 0 &{} 0 \\ 0 &{} \tau _b &{} 0 \\ 0 &{} 0 &{} \tau _e \\ \end{bmatrix} ^{-1} \begin{bmatrix} W_{ai}Z_1 &{} 0\\ -W_{bi}M_{jk} &{} 0 \\ W_{ek}Z_3 &{} -W_{ek}Z_4 \\ \end{bmatrix} \\&\le \begin{bmatrix} 0 &{} 0 &{} 0 \\ H_{ai} &{} H_{bi} &{} H_{ek} \end{bmatrix} T \begin{bmatrix} 0 &{} H_{ai}^\top \\ 0 &{} H_{bi}^\top \\ 0 &{} H_{ek}^\top \\ \end{bmatrix} + \begin{bmatrix} Z_1^\top W_{ai}^\top &{} -M_{jk}^\top W_{bi}^\top &{} Z_3^\top W_{ek}^\top \\ 0 &{} 0 &{} -Z_4^\top W_{ek}^\top \\ \end{bmatrix} T^{-1} \begin{bmatrix} W_{ai}Z_1 &{} 0\\ -W_{bi}M_{jk} &{} 0 \\ W_{ek}Z_3 &{} -W_{ek}Z_4 \end{bmatrix} \end{aligned} \end{aligned}$$

Therefore the condition (46) is satisfied if

50 $$\begin{aligned} \begin{aligned} \begin{bmatrix} -{Z}_3-{Z}_3^\top &{} * \\ {Z}_4^\top +{A}_i {Z}_1-{B}_i {M}_{j k}+{E}_k {Z}_3 &{} -{Z}_4^\top {E}_k^\top -{E}_k {Z}_4 \end{bmatrix}&+ \begin{bmatrix} 0 &{} 0 &{} 0 \\ H_{ai} &{} H_{bi} &{} H_{ek} \\ \end{bmatrix} T \begin{bmatrix} 0 &{} H_{ai}^\top \\ 0 &{} H_{bi}^\top \\ 0 &{} H_{ek}^\top \\ \end{bmatrix} \\ +&\begin{bmatrix} Z_1^\top W_{ai}^\top &{} -M_{jk}^\top W_{bi}^\top &{} Z_3^\top W_{ek}^\top \\ 0 &{} 0 &{} -Z_4^\top W_{ek}^\top \\ \end{bmatrix} T^{-1} \begin{bmatrix} W_{ai}Z_1 &{} 0\\ -W_{bi}M_{jk} &{} 0 \\ W_{ek}Z_3 &{} -W_{ek}Z_4 \\ \end{bmatrix} < 0 \end{aligned} \end{aligned}$$

Utilizing the Schur complement, the above inequality can be rewritten as:

51 $$\begin{aligned}{}&\left[ \begin{array}{cc} \begin{array}{l} \left[ \begin{array}{cc} -{Z}_3-{Z}_3^\top &{} * \\ {Z}_4^\top +{A}_i {Z}_1-{B}_i {M}_{j k}+{E}_k {Z}_3 &{} -{Z}_4^\top {E}_k^\top -{E}_k {Z}_4 \end{array}\right] + \left[ \begin{array}{ccc} 0 &{} 0 &{} 0 \\ H_{ai} &{} H_{bi} &{} H_{ek} \\ \end{array} \right] T \left[ \begin{array}{cc} 0 &{} H_{ai}^\top \\ 0 &{} H_{bi} ^\top \\ 0 &{} H_{ek}^\top \\ \end{array} \right] \end{array} &{} * \\ \left[ \begin{array}{ccc} W_{ai}Z_1 &{} 0\\ -W_{bi}M_{jk} &{} 0 \\ W_{ek}Z_3 &{} -W_{ek}Z_4 \\ \end{array} \right] &{} -\left[ \begin{array}{ccc} \tau _a &{} 0 &{} 0 \\ 0 &{} \tau _b &{} 0 \\ 0 &{} 0 &{} \tau _e \\ \end{array} \right] \\ \end{array} \right] < 0 \end{aligned}$$

52 $$\begin{aligned}{}&\Leftrightarrow \left[ \begin{array}{ccccc} -{Z}_3-{Z}_3^\top &{} * &{} * &{} * &{} *\\ {Z}_4^\top +{A}_i {Z}_1-{B}_i {M}_{j k}+{E}_k {Z}_3 &{} -{Z}_4^\top {E}_k^\top -{E}_k {Z}_4 +\tau _aH_{ai}H_{ai}^\top +\tau _bH_{bi}H_{bi}^\top +\tau _eH_{ek}H_{ek}^\top &{} * &{} * &{} *\\ W_{ai}Z_1 &{} 0 &{} -\tau _a &{} * &{} *\\ -W_{bi}M_{jk} &{} 0 &{} 0 &{} -\tau _b &{} *\\ W_{ek}Z_3 &{} -W_{ek}Z_4 &{} 0 &{} 0 &{} -\tau _e \end{array} \right] <{0} \end{aligned}$$

$$\square$$

The following theorem unifies the strengths of both optimal and robust control methodologies, demonstrating the advantages of their amalgamation in the form of robust-optimal control. This theorem asserts that by judiciously combining the principles outlined in Theorems 2 and 3, we can achieve control strategies that optimize performance while robustly handling uncertainties, thereby offering a comprehensive and versatile approach to control system design.

##### Theorem 4

The feedback gains $F_{ik}$ of the robust-optimal fuzzy controller can be obtained by solving the LMIs conditions of both Theorems 2 and 3:

53a $$\begin{aligned}{}&{Z}_1^\top ={Z}_1>{0}, \end{aligned}$$

53b $$\begin{aligned}{}&{\left[ \begin{array}{ccccc} -{Z}_3-{Z}_3^\top &{} * &{} * &{} * &{} *\\ {Z}_4^\top +{A}_i {Z}_1-{B}_i {M}_{j k}+{E}_k {Z}_3 &{} -{Z}_4^\top {E}_k^\top -{E}_k {Z}_4 +\tau _aH_{ai}H_{ai}^\top +\tau _bH_{bi}H_{bi}^\top +\tau _eH_{ek}H_{ek}^\top &{} * &{} * &{} *\\ W_{ai}Z_1 &{} 0 &{} -\tau _a &{} * &{} *\\ -W_{bi}M_{jk} &{} 0 &{} 0 &{} -\tau _b &{} *\\ W_{ek}Z_3 &{} -W_{ek}Z_4 &{} 0 &{} 0 &{} -\tau _e \end{array}\right] <{0}}, \end{aligned}$$

53c $$\begin{aligned}{}&{\left[ \begin{array}{cc} \lambda &{} {x}^\top (0) \\ {x} (0) &{} Z_1 \\ \end{array} \right] > 0}, \end{aligned}$$

53d $$\begin{aligned}{}&{\left[ \begin{array}{cccc} -{Z}_3-{Z}_3^\top &{} * &{} * &{} *\\ {Z}_4^\top +{A}_i {Z}_1-{B}_i {M}_{i k}+{E}_k {Z}_3 &{} -{Z}_4^\top {E}_k^\top -{E}_k {Z}_4 &{} * &{} *\\ C_iZ_1 &{} 0 &{} -W^{-1} &{} * \\ -M_{ik} &{} 0 &{} 0 &{} -R^{-1} \end{array}\right] <{0},}\hspace{30pt} h_i \cap v_k \ne \varnothing \text {, } \end{aligned}$$

53e $$\begin{aligned}{}&{\left[ \begin{array}{cccccc} -2 {Z}_3-2 {Z}_3^\top &{} * &{} * &{} * &{} * &{} *\\ 2 {Z}_4^\top +{A}_i {Z}_1 -{B}_i {M}_{j k}+{A}_j {Z}_1 -{B}_j {M}_{i k}+2 {E}_k {Z}_3 &{} -2 {Z}_4^\top {E}_k^\top -2 {E}_k {Z}_4 &{} * &{} * &{} * &{} *\\ C_iZ_1 &{} 0 &{} -W^{-1} &{} * &{} * &{} *\\ -M_{jk} &{} 0 &{} 0 &{} -R^{-1} &{} * &{} *\\ C_jZ_1 &{} 0 &{} 0 &{} 0 &{} -W^{-1} &{} *\\ -M_{ik} &{} 0 &{} 0 &{} 0 &{} 0 &{} -R^{-1} \\ \end{array}\right]< {0},} \nonumber \\&i<j \le r \text { s.t. } h_i \cap h_j \cap v_k \ne \varnothing \end{aligned}$$

##### Proof

It follows directly from Theorems 2 and 3. $\square$

### Applying to the RIP system

In the previous section, control design principles were detailed, establishing a comprehensive framework of advanced techniques and strategies. This section shifts the focus to their practical application, centered on the rotary inverted pendulum system. This dynamic configuration, comprising an inverted pendulum atop a rotating base, offers an intriguing platform for the implementation of the earlier control methods. Its inherent instability, nonlinearity, and susceptibility to external disturbances render it an ideal candidate for showcasing the control techniques’ capabilities. By directing our control strategies toward this intricate system, the goal is to not only stabilize and govern the rotary inverted pendulum but also to underscore the adaptability and effectiveness of the methods in addressing real-world challenges. Consider the dynamic equation of the rotary inverted pendulum system^31^:

54 $$\begin{aligned} {\left\{ \begin{array}{ll} \displaystyle \frac{4}{3} c_1 \ddot{\phi }+c_2 \ddot{\theta } \cos \phi -c_1 {\dot{\theta }}^2 \sin \phi \cos \phi +c_3 {\dot{\phi }}-c_4 \sin \phi =0 \\ c_2 \ddot{\phi } \cos \phi +\left( c_5+c_1 \sin ^2 \phi \right) \ddot{\theta }-c_2 {\dot{\phi }}^2 \sin \phi +c_6 {\dot{\theta }} +2 c_1 {\dot{\phi }} {\dot{\theta }} \sin \phi \cos \phi =c_7 V_m \end{array}\right. } \end{aligned}$$

where $\displaystyle c_1=\frac{m l^2}{4}$, $\displaystyle c_2=\frac{m l r}{2}$, $\displaystyle c_3=B_r$, $\displaystyle c_4=\frac{m g l}{2}$, $\displaystyle c_5=J_{e q}+m r^2+\eta _g K_g^2 J_m$, $\displaystyle c_6=B_a+\frac{\eta _m \eta _g K_t K_v K_g^2}{R}$, $\displaystyle c_7=\frac{\eta _m \eta _g K_t K_g}{R}$. The above coefficients can be calculated with the parameters in Table 1.

**Table 1:** RIP system parameters.

Parameters	Description
$g=9.81$	Gravity $(m/s^2)$
$m=0.125$	Mass of the pendulum rod (*kg*)
$l=0.335$	Length of the pendulum rod (*m*)
$r_a=0.215$	Length of the pendulum arm (*m*)
$J_{eq}= 3.5842 \times 10^{-3}$	Equivalent moment of inertia of the pendulum arm and gears $(kgm^2)$
$J_m= 3.87 \times 10^{-7}$	Moment of inertia of the motor rotor $(kgm^2)$
$B_a= 0.004$	Friction coefficient of the pendulum arm (*Nms*/*rad*)
$B_r= 0.0095$	Friction coefficient of the pendulum rod (*Nms*/*rad*)
$K_t= 7.67 \times 10^{-3}$	Torque constant (*Nm*/*A*)
$K_v= 7.67 \times 10^{-3}$	Back EMF constant (*Nm*/*A*)
$R= 2.6$	Motor armature resistance $(\Omega )$
$K_g= 70$	Gearbox ratio
$\eta _g= 0.9$	Gearbox efficiency
$\eta _m= 0.69$	Motor efficiency

If the premise variables are chosen as

55 $$\begin{aligned} {\left\{ \begin{array}{ll} z_1(t) &{}= cos\phi , \\ z_2(t) &{}= sin^2\phi , \\ z_3(t) &{}= \displaystyle \frac{sin\phi }{\phi },\\ z_4(t) &{}= {\dot{\theta }}sin\phi cos\phi , \\ z_5(t) &{}= {\dot{\phi }}sin\phi , \end{array}\right. } \end{aligned}$$

the matrices *A*, *B*, *E* of the fuzzy descriptor system in (1) will be:

$$\begin{aligned} E = \begin{bmatrix} 1 &{} 0 &{} 0 &{} 0 \\ 0 &{} 1 &{} 0 &{} 0 \\ 0 &{} 0 &{} \frac{4}{3}c_1 &{} c_2z_1(t) \\ 0 &{} 0 &{} c_2z_1(t) &{} c_5 + c_1z_2(t) \\ \end{bmatrix}, \quad A = \begin{bmatrix} 0 &{} 0 &{} 1 &{} 0 \\ 0 &{} 0 &{} 0 &{} 1 \\ c_4z_3(t) &{} 0 &{} -c_3 &{} c_1z_4(t) \\ 0 &{} 0 &{} c_2z_5(t)-2c_1z_4(t) &{} -c_6 \\ \end{bmatrix}, \quad B = \begin{bmatrix} 0 &{} 0 &{} 0 &{} c_7 \\ \end{bmatrix} ^\top . \end{aligned}$$

Some features can be highlighted when representing the RIP system by fuzzy descriptor system:

- It can depict a broader range of systems compared to the conventional T–S fuzzy system by mitigating the denominator term within matrix *A*;
- In^32^, the RIP system necessitates 8 premise variables for its representation in a T–S fuzzy system. However, when employing the descriptor model, only 5 variables are required;
- The selection of *z*(*t*) is considerably more straightforward in comparison to^32^, thus alleviating the computational workload in subsequent steps.

It’s evident that the *E* matrix comprises 2 premise variables, while the *A* matrix contains 3 premise variables. Consequently, on the left side, we have 4 fuzzy rules $(2^2)$ and 8 fuzzy rules $(2^3)$ on the right side. For each fuzzy rule, we determine the precise $A_i$ and $E_k$ using the following approach:

**Table 2:** Rules for determining the values of $A_i$ matrices.

	$A_1$	$A_2$	$A_3$	$A_4$	$A_5$	$A_6$	$A_7$	$A_8$
$z_3(t)$	$z_{3min}$	$z_{3min}$	$z_{3min}$	$z_{3min}$	$z_{3max}$	$z_{3max}$	$z_{3max}$	$z_{3max}$
$z_4(t)$	$z_{4min}$	$z_{4min}$	$z_{4max}$	$z_{4max}$	$z_{4min}$	$z_{4min}$	$z_{4max}$	$z_{4max}$
$z_5(t)$	$z_{5min}$	$z_{5max}$	$z_{5min}$	$z_{5max}$	$z_{5min}$	$z_{5max}$	$z_{5min}$	$z_{5max}$

**Table 3:** Rules for determining the values of $E_k$ matrices.

	$E_1$	$E_2$	$E_3$	$E_4$
$z_1(t)$	$z_{1min}$	$z_{1min}$	$z_{1max}$	$z_{1max}$
$z_2(t)$	$z_{2min}$	$z_{2max}$	$z_{2min}$	$z_{2max}$

Consider the uncertain descriptor model has the form (41), with the variation of the pendulum mass is uncertainty, the uncertain matrices $\Delta A$, $\Delta E$ can be inferred as:

$$\begin{aligned} \Delta E = \begin{bmatrix} 0 &{} 0 &{} 0 &{} 0 \\ 0 &{} 0 &{} 0 &{} 0 \\ 0 &{} 0 &{} \frac{4}{3}\Delta c_1 &{} \Delta c_2z_1(t) \\ 0 &{} 0 &{} \Delta c_2z_1(t) &{} \Delta c_5 + \Delta c_1z_2(t) \\ \end{bmatrix}, \quad \Delta A = \begin{bmatrix} 0 &{} 0 &{} 0 &{} 0 \\ 0 &{} 0 &{} 0 &{} 0 \\ \Delta c_4z_3(t) &{} 0 &{} 0 &{} \Delta c_1z_4(t) \\ 0 &{} 0 &{} \Delta c_2z_5(t)-\Delta c_1z_4(t) &{} 0 \\ \end{bmatrix}. \end{aligned}$$

From the definition of $\Delta A$ and $\Delta E$, we have:

56 $$\begin{aligned} {\left\{ \begin{array}{ll} H_{ai} = \begin{bmatrix} 0 &{} 0 \\ 0 &{} 0 \\ 0 &{} 1 \\ 1 &{} 0 \\ \end{bmatrix}, \quad \Delta a_i(t)= \begin{bmatrix} f_a(t) &{} 0 \\ 0 &{} f_a(t) \\ \end{bmatrix}, \quad W_{ai} = \begin{bmatrix} 0 &{} 0 &{} \Delta c_2z_{5m}-\Delta c_1z_{4m} &{} 0 \\ \Delta c_4z_{3m} &{} 0 &{} 0 &{} \Delta c_1z_{4m} \end{bmatrix}, \\ H_{ek} = \begin{bmatrix} 0 &{} 0 \\ 0 &{} 0 \\ 1 &{} 0 \\ 0 &{} 1 \\ \end{bmatrix}, \quad \Delta e_k(t) = \begin{bmatrix} f_e(t) &{} 0 \\ 0 &{} f_e(t) \\ \end{bmatrix}, \quad W_{ek} = \begin{bmatrix} 0 &{} 0 &{} \frac{4}{3}\Delta c_1 &{} \Delta c_2z_{1m} \\ 0 &{} 0 &{} \Delta c_2z_{1m} &{} \Delta c_5 + \Delta c_1z_{2m} \end{bmatrix} \end{array}\right. } \end{aligned}$$

where $-1\le f_a(t), f_e(t)\le 1$ and the values of $z_{1m}$, $z_{2m}$, $z_{3m}$, $z_{4m}$ and $z_{5m}$ can be chosen similarly to those in Tables 2 and 3.

Choosing the conditions $\theta \in \left[ \frac{-\pi }{2};\frac{\pi }{2}\right]$, $\phi \in \left[ \frac{-\pi }{4};\frac{\pi }{4}\right]$, ${\dot{\theta }}\in \left[ -10;10\right]$, ${\dot{\phi }}\in \left[ -10;10\right]$, the boundary of premise variables can be calculated as shown in table 4:

**Table 4:** Boundary of premise variables.

	$z_1(t)$	$z_2(t)$	$z_3(t)$	$z_4(t)$	$z_5(t)$
Maximum	0.7071	0	0.9003	$-$0.75	$-$7.0711
Minimum	1	0.5	1	0.75	7.0711

From the above maximum and minimum values, weighting functions for each premise variable are derived:

57 $$\begin{aligned} \zeta ^1_i = \frac{z_{imax}-z_i(t)}{z_{imax}-z_{imin}}, \quad \zeta ^0_i = 1 - \zeta ^1_i, \quad i = \{1,2,3,4,5\}. \end{aligned}$$

Therefore, the membership functions satisfying the convex sum property are determined:

$$\begin{aligned} v_1(z(t))&= \zeta ^0_1*\zeta ^0_2, \quad v_2(z(t)) = \zeta ^0_1*\zeta ^1_2, \\ v_3(z(t))&= \zeta ^1_1*\zeta ^0_2, \quad v_4(z(t)) = \zeta ^1_1*\zeta ^1_2, \\ h_1(z(t))&= \zeta ^0_3*\zeta ^0_4*\zeta ^0_5, \quad h_2(z(t)) = \zeta ^0_3*\zeta ^0_4*\zeta ^1_5, \\ h_3(z(t))&= \zeta ^0_3*\zeta ^1_4*\zeta ^0_5, \quad h_4(z(t)) = \zeta ^0_3*\zeta ^1_4*\zeta ^1_5, \\ h_5(z(t))&= \zeta ^1_3*\zeta ^0_4*\zeta ^0_5, \quad h_6(z(t)) = \zeta ^1_3*\zeta ^0_4*\zeta ^1_5, \\ h_7(z(t))&= \zeta ^1_3*\zeta ^1_4*\zeta ^0_5, \quad h_8(z(t)) = \zeta ^1_3*\zeta ^1_4*\zeta ^1_5. \end{aligned}$$

## Simulation and discussion

In continuation of the theoretical groundwork elucidated in Section “Control design”, this section embarks on the practical implementation and validation of the control methodologies through three distinct simulation scenarios:

- Case 1 (Stability control): In this scenario, we delve into the realm of stability control, evaluating the efficacy of our control strategies in maintaining the equilibrium and stability of the system.
- Case 2 (Optimal control): The second scenario delves into optimal control, where we explore how our control techniques can be harnessed to optimize system performance and achieve desired objectives.
- Case 3 (Robust-Optimal Control): The third scenario combines the principles of robust and optimal control, demonstrating how our unified control approach copes with uncertainties and disturbances while optimizing system performance.

**Figure 1:**
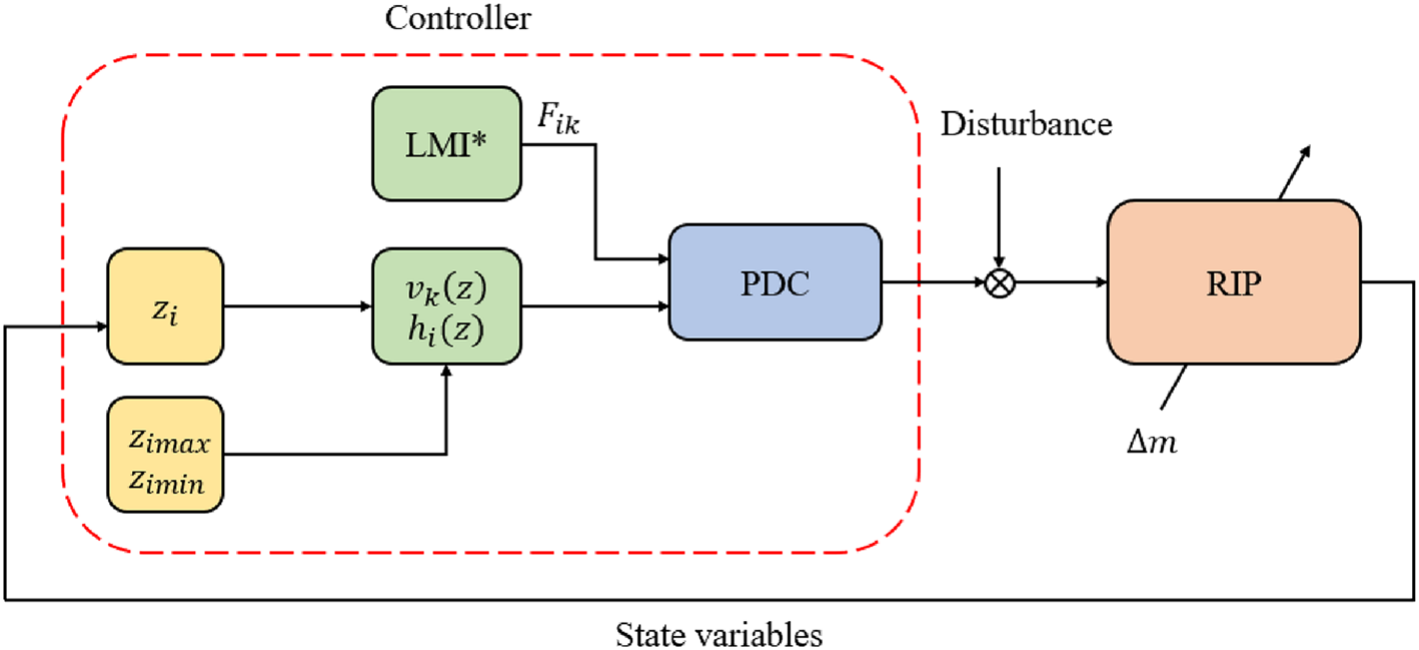
Control structure (*LMI condition for each case: Stability control, Optimal control, Robust-Optimal control).

These simulation scenarios collectively serve as a comprehensive testbed, allowing us to assess the practical applicability and versatility of our control methodologies in various operational contexts. For a visual representation of the simulation scenarios, Fig. 1 displays the control structure of the system across all three cases. Drawing from the previously outlined theories, varied linear matrix inequalities computations result in diverse control matrices. The instantaneous values of premise variables and membership functions are calculated by integrating feedback from state variables. Subsequently, all these derived values are consolidated according to the PDC law to construct the comprehensive controller.

### Case 1: stability control

In this instance, the utilization of a stability control theory-based controller is planned. To demonstrate the control method’s effectiveness and analyze output responses under varying conditions, three distinct initial conditions will be introduced.

**Figure 2:**
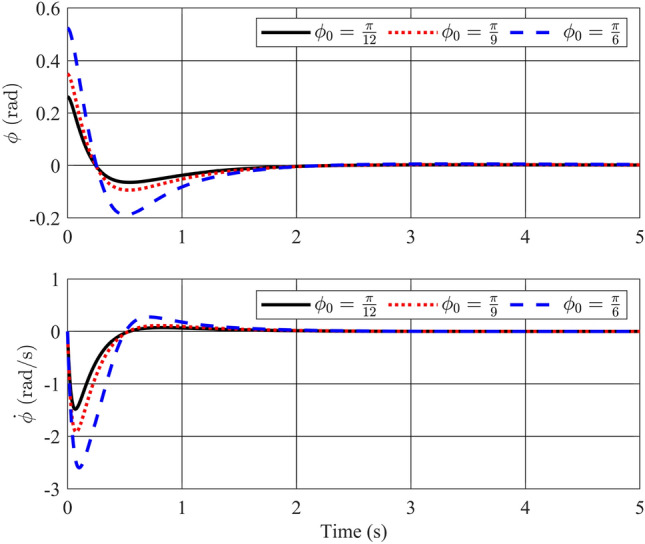
Angle of the pendulum and its velocity with stability controller.

**Figure 3:**
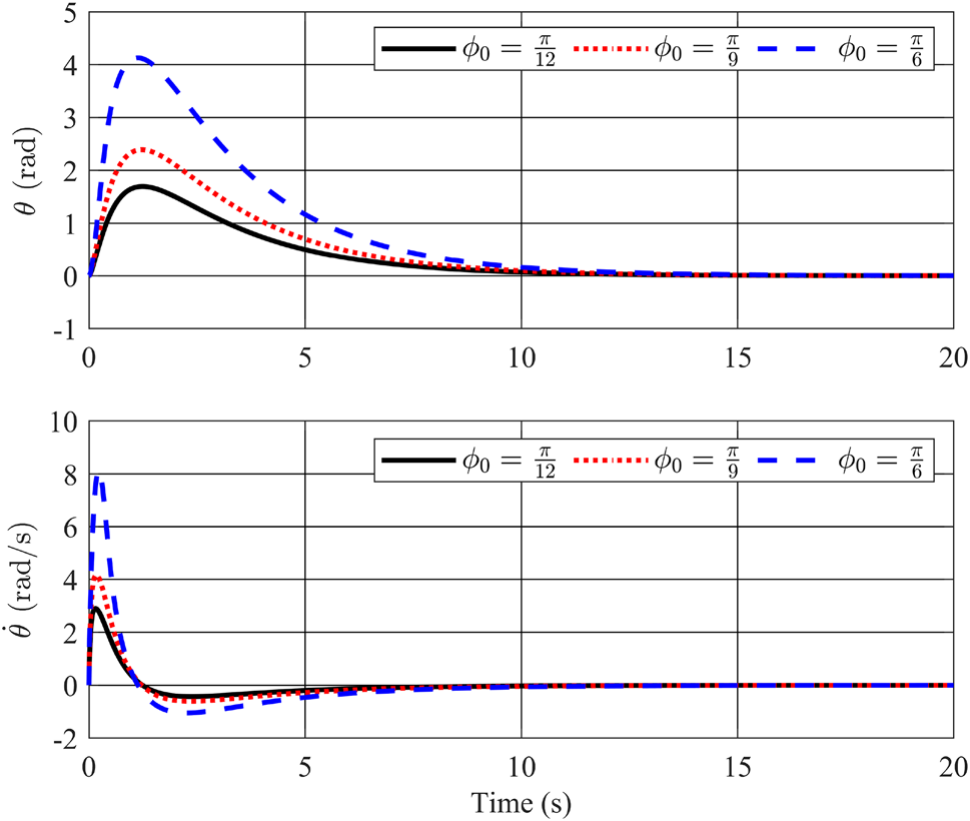
Angle of the rotary arm and its velocity with stability controller.

Figures 2 and 3 depict the trajectories of the four state variables across three distinct scenarios, each corresponding to different initial deflection angles of the pendulum rod: $\frac{\pi }{12}$(rad),$\frac{\pi }{9}$(rad), and $\frac{\pi }{6}$(rad), respectively. Examining the pendulum’s initial deflection angles, it’s evident from the graphs that the stabilization controller performs effectively when the initial angles are relatively modest, swiftly restoring the pendulum to an upright and stable position. Notably, larger initial angles result in greater pendulum oscillations and extended settling times. Nevertheless, the variance in settling times remains relatively minor, all hovering around the 2-second mark. The angular velocity of the pendulum also converges to zero as the pendulum bar reaches the desired position. On the other hand, state variables related to the pendulum arm, such as arm position ($\theta$) and arm angular velocity (${\dot{\theta }}$), exhibit longer settling times. Specifically, for initial angles of 15, 20, and 30 degrees, the time required for the arm angle to return to zero measures 10.82s, 11.66s, and 12.95s, respectively. Although these scenarios achieve stable equilibrium positions for both position and velocity, there remains room for enhancing the overall system’s settling time. In summary, it is apparent that the utilization of a fuzzy descriptor-based model and LMI-based control design effectively achieves stability for the rotary inverted pendulum, fulfilling the fundamental control requirements of the system.

**Figure 4:**
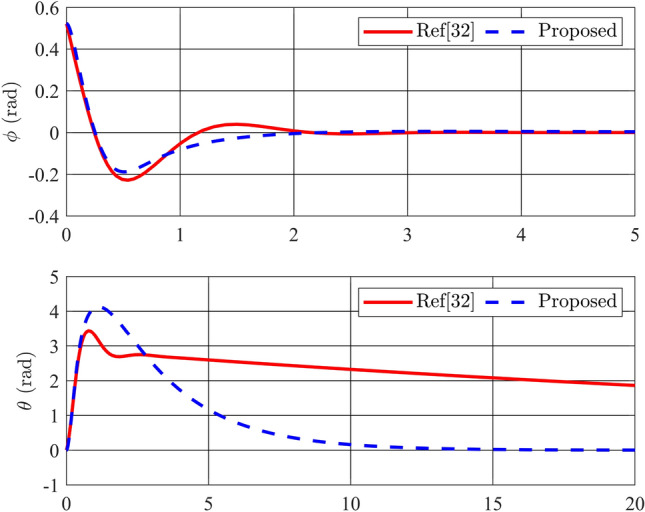
Angle of the pendulum and its velocity in comparison between Ref^32^ and proposed stability controller.

For a comprehensive assessment of the proposed control method, a comparison is conducted between our stability controller and the one outlined in reference^32^. The simulation results depicted in Fig. 4 showcase the performance of the two controllers when subjected to the same initial angle condition of 30 degrees. It is evident that the angular responses of the pendulum bar for both controllers exhibit similar quality, converging to zero after approximately 2 s. The controller investigated in the referenced study even displays superior steady-state error values for the variable $\phi$. However, a contrasting scenario unfolds for variables related to the pendulum arm: the settling time of the arm angle controlled by the referenced controller is significantly longer than that of the proposed controller. This comparison highlights that despite both controllers utilizing PDC for fuzzy systems, differences in system representation and the structure of their LMIs can lead to distinct control signals and markedly different system responses.

#### Case 2: optimal control

As previously discussed in earlier sections and the analysis of simulation results in Case 1, there is a clear need for enhancement in the overall system’s response time, particularly concerning the settling time of the two variables, $\theta$ and ${\dot{\theta }}$. Furthermore, it is imperative to consider the cumulative cost function value throughout the entirety of the simulation, as it serves as a comprehensive metric for evaluating the system’s overall efficiency and performance.

**Figure 5:**
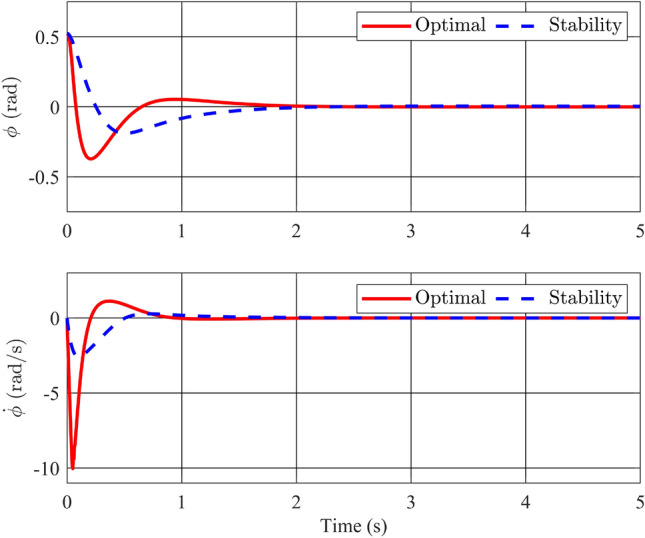
Angle and its velocity of the pendulum in the comparison of optimal and stability controllers.

In Fig. 5, an initial condition of $\phi _0=\frac{\pi }{6}$(rad) is imposed on both control methodologies. Notably, given that the optimal controller is constructed upon the principles of a stable controller, the angular response of the pendulum bar exhibits stability, converging to the equilibrium position. Remarkably, the red line, corresponding to the optimal controller, demonstrates a setup time of merely 2 s, slightly outpacing the blue line. While the maximum deflection angle is marginally larger when using fuzzy optimal controller, the steady-state error is nearly imperceptible. Achieving this swift and precise angular response inevitably entails more rapid and substantial angular velocity changes compared to when employing a stabilization controller. However, it is worth noting that the elevated angular velocity of the pendulum bar does not exert a significant impact on the system’s structural integrity and is deemed acceptable.

**Figure 6:**
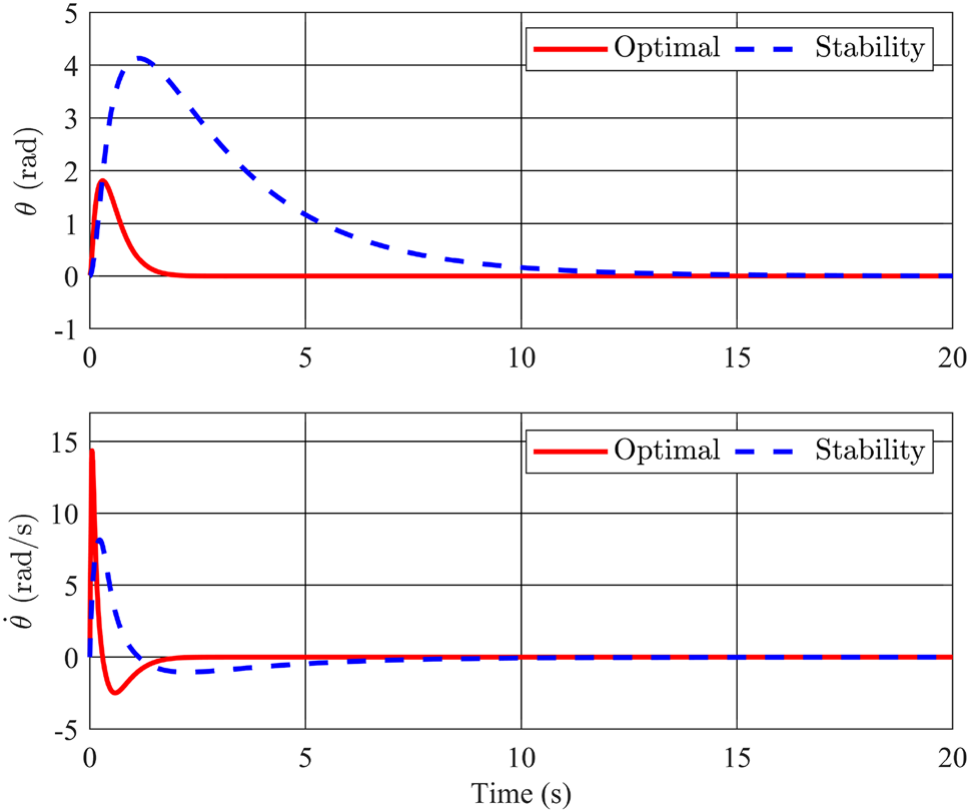
Angle and its velocity of the rotary arm in the comparison of optimal and stability controllers.

The most noteworthy enhancement is readily apparent in the responses of the pendulum arm, as depicted in Fig. 6. Consider the angular position variable $\theta$: not only is the oscillation amplitude significantly diminished, decreasing from 4.13 rad to a mere 1.81 rad, but the settling time has also undergone a remarkable reduction of over sixfold, plummeting from a lengthy 13 s to approximately 2 s. The utilization of the optimal controller effectively mitigates the drawbacks associated with the original stable controller while further refining the oscillation amplitude. Consequently, the angular velocity of the pendulum arm is relatively substantial and exhibits a sharp peak, indicative of heightened motor acceleration. This heightened acceleration necessitates more robust experimental equipment to accommodate the increased demands. Considering the cost function *J* in (21) over a simulation time of 20s, we have:

58 $$\begin{aligned} J=\int _{0}^{20}\left( {y}^\top (t) \begin{bmatrix} 1 &{} 0 &{} 0 &{} 0 \\ 0 &{} 1 &{} 0 &{} 0 \\ 0 &{} 0 &{} 1 &{} 0 \\ 0 &{} 0 &{} 0 &{} 1 \\ \end{bmatrix} y(t)+u^\top (t)u(t) \right) dt. \end{aligned}$$

Following the completion of the simulation process, the total calculated costs associated with the optimal controller and the conventional stability controller amount to 58.4354 and 81.8599, respectively. This comparison clearly illustrates that the optimal controller not only fulfills the fundamental requirement of stabilizing the inverted pendulum system but also contributes to a notable reduction in the overall system cost.

#### Case 3: robust optimal control

In Case 3, we delve into the incorporation of the model’s uncertainty components to assess and compare the performance of both the optimal and robust-optimal controllers. This investigation will encompass two distinct simulation scenarios. In the first scenario, we introduce a modification by altering the mass of the pendulum bar, specifically by $\Delta m = 0.25m$. This $\Delta m$ value serves to determine the uncertainty components in equation (56), which encompass $\Delta c_1$, $\Delta c_2$,..., $\Delta c_5$. Consequently, this enables the determination of the matrices $H_{ai}$ and $W_{ai}$, which are subsequently incorporated into the LMI condition (43b) for computational purposes. In the second scenario, we inject white noise disturbance into the control input, mirroring the uncertainty embedded within the control matrix *B*.

**Figure 7:**
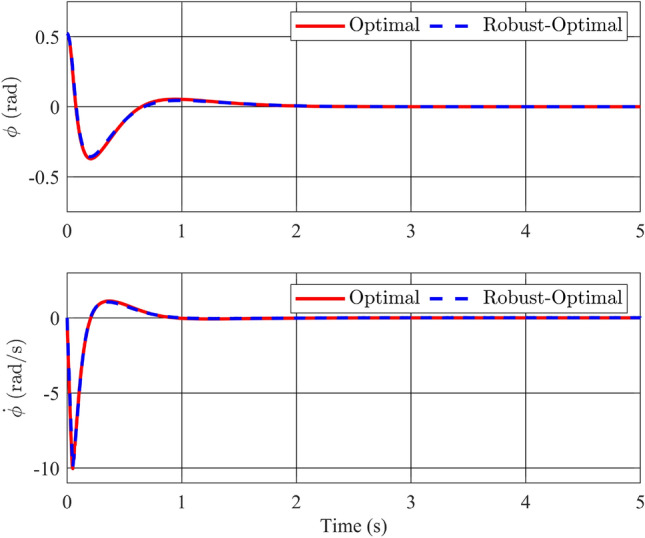
Angle and its velocity of the pendulum in the comparison of optimal and robust-optimal controllers in scenario 1.

**Figure 8:**
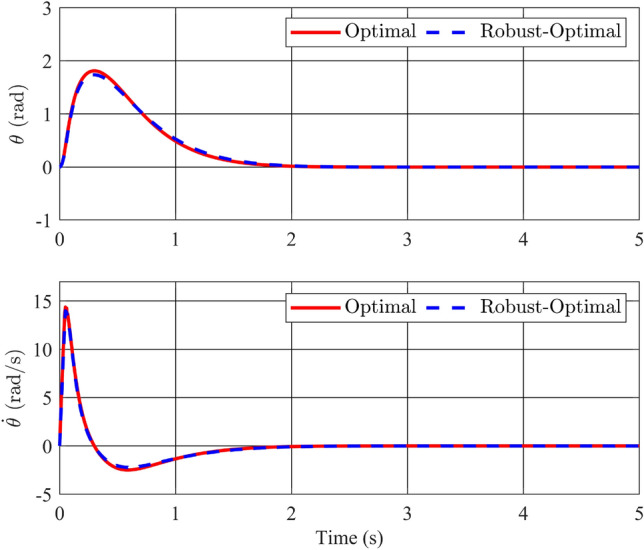
Angle and its velocity of the rotary arm in the comparison of optimal and robust-optimal controllers in scenario 1.

As depicted in Figures 7 and 8, the response profiles of the state variables remain remarkably consistent between the two controllers in the first scenario, wherein the mass of the pendulum bar undergoes a controlled variation. Significant differences in amplitude or settling time are absent, with both controllers effectively guiding the pendulum to its desired position in approximately 2 s. The peak angular velocity of the pendulum bar does not significantly diverge between the controllers, remaining at 10 rad/s and steadily approaching zero within the initial second. Likewise, the positional response of the rotating arm exhibits minimal variance, as both controllers effectively converge this value to nearly zero within 2 s. Notably, the optimal controller registers a slightly larger peak value for the arm angle compared to the robust-optimal controller, recording 1.85 rad and 1.8 rad, respectively. In terms of angular velocity for the rotating arm, the blue and red response lines closely overlap, with both controllers exhibiting peak values of approximately ${{\dot{\phi }}} = 14$ rad/s. This velocity range equations with the capabilities of most existing motors, thereby rendering the application of this control algorithm to experimental models highly feasible. It can be observed that all state variables of the inverted pendulum system converge to their desired equilibrium positions. This outcome can be attributed to the intrinsic nonlinear characteristics of the fuzzy controller, which inherently exhibits a degree of resistance to model variations, including uncertainties. Notwithstanding, the discernible disparities between the optimal controller and the robust-optimal controller become more pronounced when the uncertainties under consideration are subjected to significant and unpredictable fluctuations. Therefore, the second scenario will explore conditions where uncertainties are not only present but also constantly changing.

**Figure 9:**
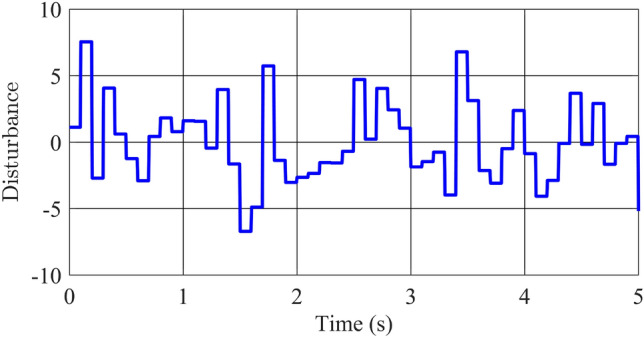
White noise disturbance.

In this second scenario, alongside the modification of the pendulum bar’s mass, we introduce a white noise signal as shown in Fig. 9 into the control signal. Consequently, this noise is integrated into matrix *B* as an uncertain component of the model.

**Figure 10:**
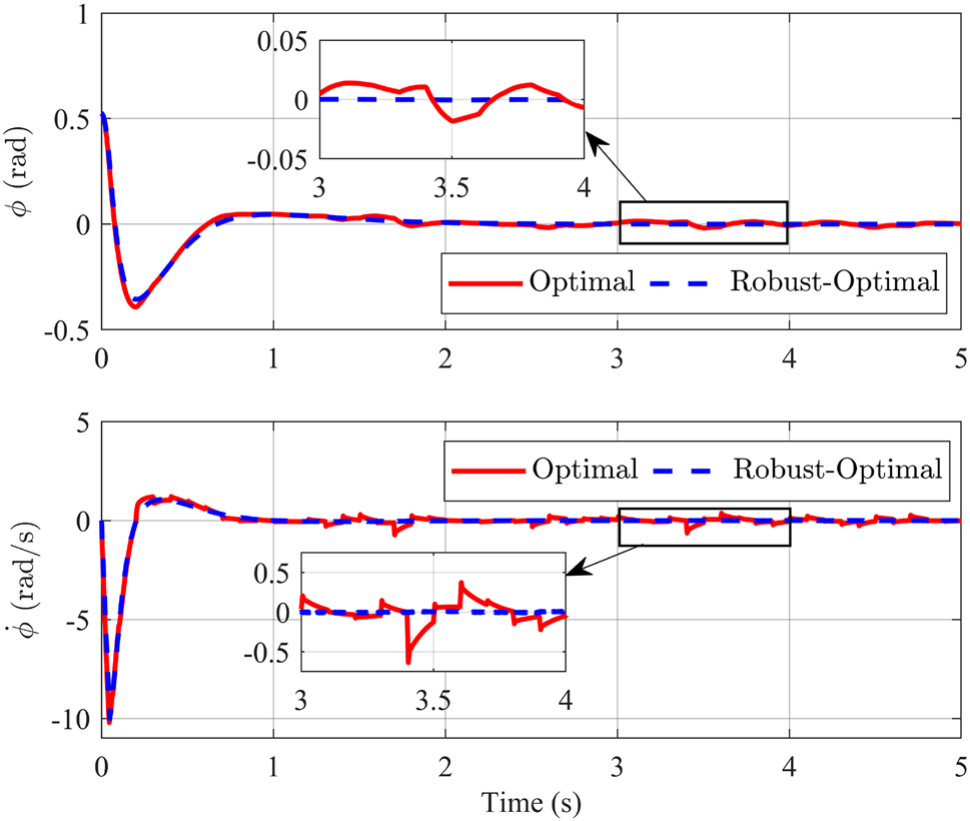
Angle and its velocity of the pendulum in the comparison of optimal and robust-optimal controllers in scenario 2.

**Figure 11:**
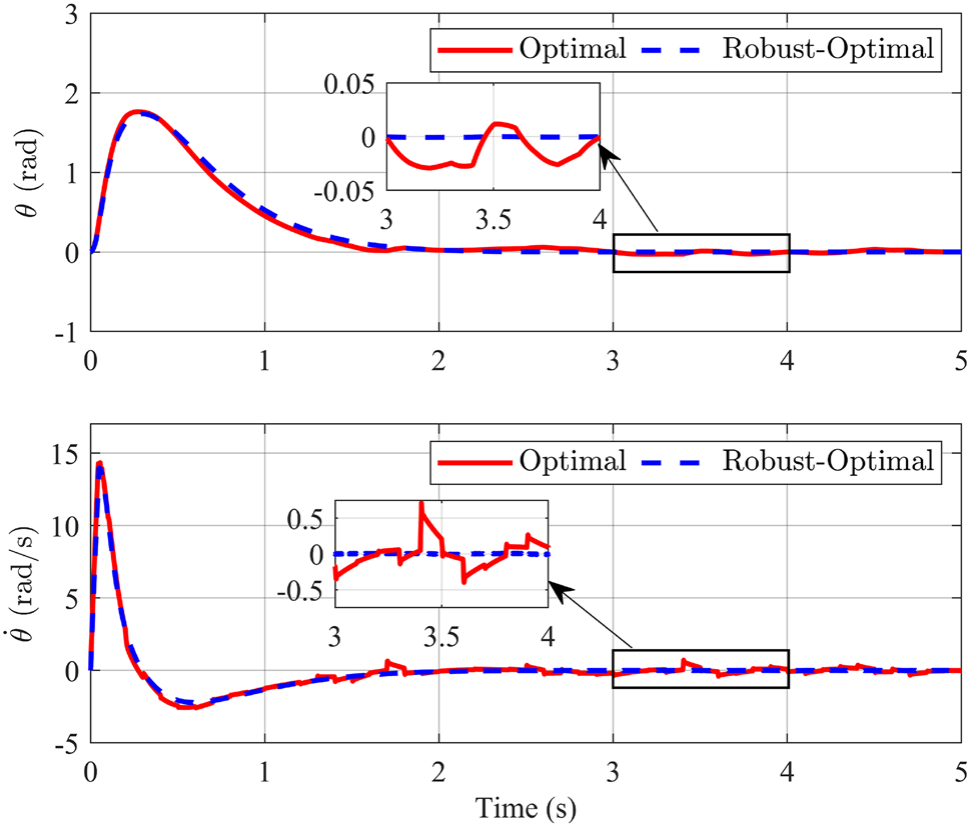
Angle and its velocity of the rotary arm in the comparison of optimal and robust-optimal controllers in scenario 2.

**Figure 12:**
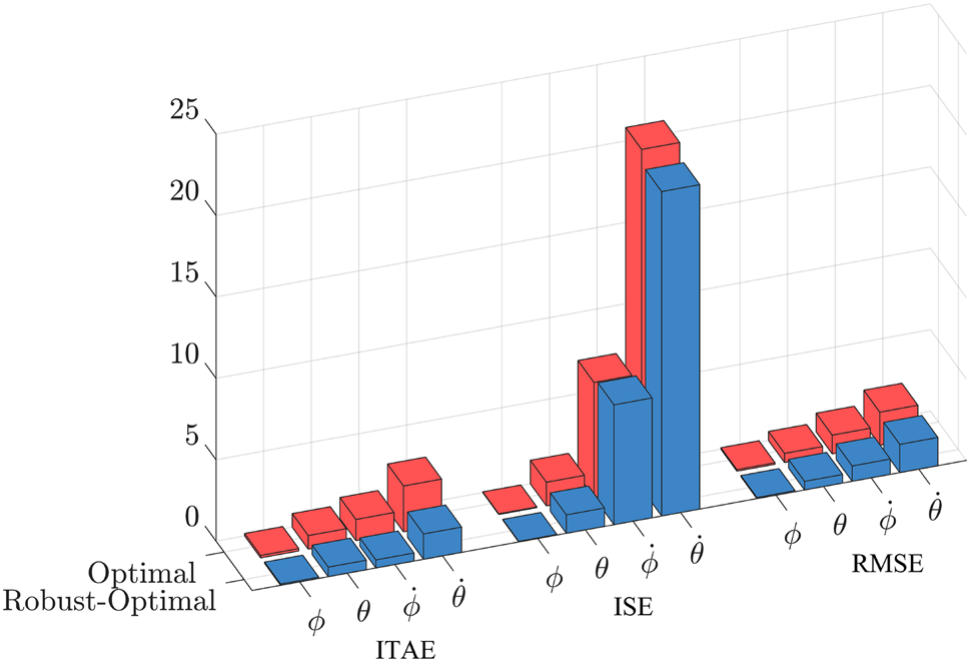
Comparison criteria between two controllers.

When examining the angular responses and angular velocities depicted in Fig. 10, the evident superiority of the robust-optimal controller over the optimal controller becomes apparent. When subjected to variable noise, both controllers effectively maintain pendulum stability at the desired position within approximately 2 s, a result consistent with the first scenario. However, a notable distinction arises in their responses. Specifically, when the robust-optimal control is employed, the pendulum bar exhibits virtually unchanged oscillation angles during steady-state operation, whereas the fuzzy optimal controller introduces clear oscillations. Although the pendulum bar’s maximum oscillation amplitude in steady state merely reaches 0.0175 rad, this nonetheless induces minor vibrations. Similar observations apply to the responses of the rotating arm bar detailed in Fig. 11. To facilitate a more intuitive comparison between the two controllers in this simulation scenario, Table 5 and Fig. 12, provided below, furnishes the Root Mean Square Error (RMSE), Integral Time Absolute Error (ITAE) and Integral Square Error (ISE) values for the state variables governed by both controllers.

**Table 5:** Comparison standard with two controllers.

Comparison standard	Controller	$\phi$ (rad)	$\theta$ (rad)	${\dot{\phi }}$ (rad/s)	${\dot{\theta }}$ (rad/s)
RMSE	Optimal	0.0932	0.5571	1.1487	2.0264
	Robust-Optimal	0.0707	0.5308	0.9445	1.7372
ITAE	Optimal	0.1503	0.8377	1.2953	2.8179
	Robust-Optimal	0.0799	0.5888	0.4999	1.5601
ISE	Optimal	0.0425	1.4492	7.0451	20.8212
	Robust-Optimal	0.0414	1.1342	7.3591	19.9212

Across all state variables, it becomes evident that the error values associated with the robust-optimal controller are consistently smaller in magnitude than those observed under the optimal controller’s governance. This outcome substantiates the robust-optimal controller’s superior capacity in maintaining precision and minimizing error across the state variables, further affirming its resilience in the face of dynamic uncertainties.

## Conclusions

In this comprehensive study, we have introduced and explored the intricate domain of fuzzy descriptor systems, developing and rigorously analyzing three distinct controllers: the stability controller, optimal controller, and robust-optimal controller. These controllers were subsequently applied to address the intricate dynamics of a rotary inverted pendulum system, which we initially modeled as a fuzzy descriptor system. The outcomes of our extensive simulations provide compelling insights. All three controllers demonstrated their remarkable capability to effectively stabilize the pendulum system. Notably, the optimal controller emerged as a noteworthy advancement over the stability controller, exhibiting superior performance in terms of settling time and overall cost reduction. However, the robust-optimal controller further heightened the system’s resilience, particularly in the face of continuously changing uncertainties. This extensive exploration underscores the adaptability and potential of these controllers in practical applications, particularly those characterized by dynamic and uncertain environments. Moreover, the model parameters are aligned with the Quanser model specifications, establishing a basis for practical application. Subsequent research will delve into the system’s dynamics in the discrete time domain, enhancing its relevance for real-world implementation. Ultimately, this paper contributes valuable knowledge to the realm of control theory and robotics, offering a foundation for more advanced and robust control strategies in complex systems.

## Data Availability

The datasets used and/or analyzed during the current study are available from the corresponding author upon reasonable request.
